# Compromised NMDA/Glutamate Receptor Expression in Dopaminergic Neurons Impairs Instrumental Learning, But Not Pavlovian Goal Tracking or Sign Tracking^1^,^2^,^3^

**DOI:** 10.1523/ENEURO.0040-14.2015

**Published:** 2015-06-10

**Authors:** Alex S. James, Zachary T. Pennington, Phu Tran, James David Jentsch

**Affiliations:** 1 Department of Psychology, University of California, Los Angeles, Los Angeles, California 90095-1563; 2 Department of Psychiatry and Biobehavioral Sciences, University of California, Los Angeles, Los Angeles, California 90095-1563; 3 Brain Research Institute, University of California, Los Angeles, Los Angeles, California 90095-1563

**Keywords:** dopamine, incentive, learning, motivation, reward, ventral midbrain

## Abstract

Two theories regarding the role for dopamine neurons in learning include the concepts that their activity serves as a (1) mechanism that confers incentive salience onto rewards and associated cues and/or (2) contingency teaching signal reflecting reward prediction error. While both theories are provocative, the causal role for dopamine cell activity in either mechanism remains controversial. In this study mice that either fully or partially lacked NMDARs in dopamine neurons exclusively, as well as appropriate controls, were evaluated for reward-related learning; this experimental design allowed for a test of the premise that NMDA/glutamate receptor (NMDAR)-mediated mechanisms in dopamine neurons, including NMDA-dependent regulation of phasic discharge activity of these cells, modulate either the instrumental learning processes or the likelihood of pavlovian cues to become highly motivating incentive stimuli that directly attract behavior. Loss of NMDARs in dopamine neurons did not significantly affect baseline dopamine utilization in the striatum, novelty evoked locomotor behavior, or consumption of a freely available, palatable food solution. On the other hand, animals lacking NMDARs in dopamine cells exhibited a selective reduction in reinforced lever responses that emerged over the course of instrumental learning. Loss of receptor expression did not, however, influence the likelihood of an animal acquiring a pavlovian conditional response associated with attribution of incentive salience to reward-paired cues (sign tracking). These data support the view that reductions in NMDAR signaling in dopamine neurons affect instrumental reward-related learning but do not lend support to hypotheses that suggest that the behavioral significance of this signaling includes incentive salience attribution.

## Abstract (precis)

Behavior is shaped to a dramatic degree by the occurrence of rewards, through both pavlovian and instrumental conditioning processes; these mechanisms give rise to both normal and abnormal behavior. It is crucial to understand the neural mechanisms that give rise to normal actions and how they lead to pathological behaviors, such as overeating and drug addictions.

## Significance Statement

Behavior is shaped to a dramatic degree by the occurrence of rewards, through both pavlovian and instrumental conditioning processes; these mechanisms give rise to both normal and abnormal behavior. It is crucial to understand the neural mechanisms that give rise to normal actions and how they lead to pathological behaviors, such as overeating and drug addictions. Though dopamine neurotransmission has often been implicated in reward-related learning, the specifics of this role remain poorly understood. The set of studies described in this manuscript reveals that NMDA/glutamate-mediated dopamine transmission contributes to the acquisition of instrumental reward-seeking actions, possibly highlighting these mechanisms as targets of interventions designed to alter the occurrence of reward-related actions, like drug seeking and drug taking.

## Introduction

The electrical activity of dopamine neurons, and associated activity-dependent synaptic release of dopamine, is thought to be critical to reward-related learning and behavior (Wise and Rompre, 1989; Robbins and Everitt, 1992; Robinson and Berridge, 1993; Salamone, 1994; Schultz et al., 1997; Redgrave et al., 1999; Kelley, 2004). Because alterations in reward-related behaviors are found in a range of psychiatric conditions (Robinson and Berridge, 1993; Taylor and Jentsch, 2001; Neuringer, 2002; Everitt and Robbins, 2005; Martin-Soelch et al., 2007; Flagel et al., 2009; Groman et al., 2009; Shiflett and Balleine, 2011), and because many of these disorders are thought to involve dopaminergic dysfunction (Swerdlow and Koob, 1987; Billstedt, 2000; Robinson and Berridge, 2000; Everitt and Robbins, 2005; Nestler and Carlezon Jr, 2006; Iversen et al., 2008; Groman et al., 2009), understanding the mechanistic role for dopamine release in reward-driven learning remains an important research question.

A considerable body of evidence, derived mostly from electrophysiological recordings of midbrain neurons in nonhuman primates, implicates brief event-related, high-frequency discharge activity of dopaminergic neurons, and the associated phasic, nonlinear increases in the quantity of transmitter released (Grace and Bunney, 1984; Gonon, 1988; Bean and Roth, 1991), as a neural instantiation of the “prediction error” signal that figures in both classical and modern mathematical learning models (Rescorla and Wagner, 1972; Schultz et al., 1993, 1997; Sutton and Barto, 1998; Day et al., 2007). Phasic aspects of dopamine signaling may represent the difference between predicted and actually received rewards (Schultz, 2002), information used in these models to update expectancies of the organism as it learns the contingent relationships between stimuli that predict biologically significant outcomes, and the responses that produce them.

An alternate perspective regards dopaminergic transmission as the mechanism by which rewarding events and reward-predictive stimuli are imbued with incentive motivational properties, transforming them from merely pleasurable, or “liked,” to “wanted” attractors of motivated behavior and attention (Crow, 1976; Robinson and Berridge, 1993; Berridge and Robinson, 1998). A variety of lines of evidence support this conclusion: elevating dopamine release, in multiple contexts, can invigorate motivation to engage in a behavior, without affecting learning of the behavior itself (Robbins, 1978; Wyvell and Berridge, 2000, 2001; Salamone et al., 2001, 2005; Peciña et al., 2003; Cagniard et al., 2006; Yin et al., 2006). Altering dopamine can alter the magnitude of established responding immediately (Berridge, 2007), indicating that dopamine can impact reward-driven behavior without an experience of a prediction error as a precondition; indeed, aspects of reward learning are possible when dopamine is nearly absent altogether (Cannon and Palmiter, 2003; Hnasko et al., 2005), suggesting that dopamine might function to instruct motivational value, rather than associative contingencies.

The prediction error and the incentive salience perspectives are often both supported by the results of experimental manipulations of dopamine transmission. For example, optogenetic simulation of dopamine neuron burst firing acts as an unconditioned stimulus that reinforces instrumental and pavlovian behaviors (Tsai et al., 2009; Witten et al., 2011). While this establishes a causal role for phasic dopaminergic activity in reward-related learning, whether it conveys a prediction error signal that teaches contingencies or whether it instructs the incentive motivation to engage in these behaviors cannot readily be distinguished. However, studies of individual differences in the nature of behaviors expressed during autoshaping may offer a unique paradigm better suited for distinguishing these theories. Specifically, contingency learning via prediction error signals, expressed as a pavlovian approach to a reward-delivery location (goal tracking), can be differentiated from contingency learning that additionally involves incentive salience attribution to reward-predictive cues (sign tracking; Robinson and Flagel, 2009). Recent evidence suggests that the magnitude of cue-evoked, phasic dopamine release positively relates to incentive salience attribution (Flagel et al., 2011): sign-tracking rats exhibited greater conditional stimulus (CS)-elicited dopamine transients than goal trackers.

Because NMDA/glutamate receptors (NMDARs localized within midbrain dopaminergic neurons regulate dopamine transmission, including through influences on the burst firing activity; Suaud-Chagny et al., 1992), phasic dopamine release is attenuated in a mouse model lacking NMDAR in dopamine neurons (Zweifel et al., 2009; Luo et al., 2010). One application of this system, therefore, is to evaluate the effects of quantitiative reductions of afferent input-generated phasic dopamine signaling on behavior. Here, we assessed instrumental learning (which involves both prediction error and incentive salience attribution) in mice lacking NMDAR in dopamine neurons, and then studied sign-tracking/goal-tracking behavior to test the idea that NMDA-dependent aspects of dopamine signaling are causally related to propensity for incentive salience attribution.

## Materials and Methods

### Mouse lines

B6.SJL-*Slc6a3^tm1.1(cre)Bkmn^*/J (stock #006660; http://jaxmice.jax.org/strain/006660.html; referred to here as DATcre+) mice, each heterozygous for a mutated dopamine transporter (DAT) gene expressing Cre recombinase, and B6.129S4-^Grin1tm2Stl/^J (stock #005246; http://jaxmice.jax.org/strain/005246.html; referred to here as NR1*^flox/flox^*) mice were purchased from The Jackson Laboratory. In DATcre+ mice, Cre recombinase cDNA was inserted into the 3' untranslated region of the DAT gene for bicistronic mRNA translation; Cre-mediated recombination is detectable in this line as early as E15 and is primarily restricted to the substantia nigra, ventral tegmental area, and retrorubral field within the midbrain (Bäckman et al., 2006). NR1*^flox/flox^* mice have a loxP site between exons 11 and 12 and another loxP site, along with a neomycin resistance gene, at the 3' end of the *Grin1* gene (Tonegawa et al., 1996). The NR1 gene is an obligatory component of the functional NMDAR (Forrest et al., 1994), which regulates NMDAR-mediated plasticity and also dopamine cell burst firing, the latter by facilitating temporal summation of excitatory inputs (Suaud-Chagny et al., 1992; Overton and Clark, 1997). Conditional deletion of NR1 expression blocks NMDAR activity (Tsien et al., 1996), reducing the magnitude of phasic dopamine release events to ∼30% of control levels (Zweifel et al., 2009; Parker et al., 2010).

Male DATcre+ mice were bred with female NR1*^flox/flox^* mice; the DATcre+ males in the resulting F1 generation were further bred with a different set of female NR1*^flox/flox^* mice to create DATcre–;NR1*^flox/wt^*, DATcre–;NR1*^flox/flox^*, DATcre+;NR1*^flox/wt^*, and DATcre+;NR1*^flox/flox^* mice (collectively referred to as DATcre;NR1 mice). Male DATcre+ mice were also separately crossed to female B6.129S4-*GT(ROSA)26Sor^tm1Sor^*/J (stock #003474; http://jaxmice.jax.org/strain/003474.html; referred to as ROSA26-LacZ) reporter mice (Soriano, 1999), obtained from Dr. Alcino Silva’s laboratory at University of California, Los Angeles. DATcre, NR1, and ROSA26-LacZ zygosity was determined using conventional PCR methods.

Mice were between 60 and 120 d old when involved in this study. All subjects were socially housed in cages of two to four individuals with Sani-Chip cage bedding (PJ Murphy Forest Products) in a temperature- and humidity-controlled room on a 14/10 h light/dark cycle. Behavioral testing was conducted during the light cycle. Food was available *ad libitum* during locomotor behavior and free-reward consumption testing, but was restricted during other experiments, as detailed below. All animal procedures are performed according to the regulations of the university animal care committee for each author.

### LacZ X-Gal staining

DATcre+ mice also expressing the ROSA26-LacZ gene were killed by isoflurane overdose, then transcardially perfused with freshly mixed, cold 4% paraformaldehyde. Brains were stored in paraformaldehyde for 1 d before being switched to a 30% sucrose/PBS solution. Slices of 40 µm width were cut on a cryostat and rinsed in PBS. The staining solution contained 85.33 mg potassium ferrocyanide, 64 mg potassium ferricyanide, 4 ml of 20 mm MgCl_2_, 36 ml PBS, 60 mg X-gal, and 800 µl dimethylformamide. The solution was allowed to react with brain slices at 37°C for 48 h; the slices were then rinsed, counterstained, and mounted on slides.

### Quantification of monoamine utilization in the striatum

Thirty-five conscious DATcre;NR1 mice (males and females, DATcre–;NR1*^flox/wt^*, *n* = 9; DATcre–;NR1*^flox/flox^*, *n* = 8; DATcre+;NR1*^flox/wt^*, *n* = 10; DATcre+;NR1*^flox/flox^*, *n* = 8) were killed by rapid decapitation and tissue samples were collected from the ventral striatum. Samples were frozen for subsequent analyses of monoamines and their metabolites using HPLC. Tissue was homogenized in 0.1 m perchloric acid, centrifuged for 25 min, and the content of 200 μl of supernatant was quantified by reverse-phase column HPLC (BAS) at 0.7 V applied, using a 7% acetonitrile-based mobile phase. Protein content was quantified using the Lowry method (Lowry et al., 1951).

### Locomotor activity in a novel context

The locomotor behavior of 165 DATcre;NR1 mice (males and females, DATcre–;NR1*^flox/wt^*, *n* = 42; DATcre–;NR1*^flox/flox^*, *n* = 42; DATcre+;NR1*^flox/wt^* , *n* = 40; DATcre+;NR1*^flox/flox^*, *n* = 41) was characterized by placing subjects in clean, standard acrylic animal cages that were novel to the mouse (24 × 40 cm), with a thin layer of bedding. Each cage was equipped with Opto M3 locomotor activity monitors (Columbus Instruments) fitted with 1” spaced *x*-axis infrared beam emitters. Locomotor behavior was monitored for 30 min (data collected in 5 min time bins). Locomotor data for 36 mice was lost because of equipment failure, leaving *n* = 36, *n* = 32, *n* = 31, and *n* = 31 for the four genotype groups, respectively.

### Free consumption of a palatable food

Subsequently, the same sample of 165 mice used in the locomotor experiment underwent habituation to a two bottle, free-choice palatable food consumption procedure over the course of 2 d. In 2 h sessions of individual housing, mice had access to 2 Lixit tube-equipped water bottles, one filled with water and the other filled with a 10% v/v sweetened condensed milk solution (Kroger). Bottle positions (i.e., left side of the cage vs right side, order counterbalanced across genotypes) were switched on the second day of habituation. Testing began the following day, bottle positions were again switched, and data were collected for 2 d; a final switch, followed by 2 d of data collection, concluded the procedure. Data presented are averages of consumption levels on the second day of placement on each side.

### Instrumental conditioning

An experimentally naive set of 112 mice (males only, DATcre–;NR1*^flox/wt^*, *n* = 26; DATcre–;NR1*^flox/flox^*, *n* = 27; DATcre+;NR1*^flox/wt^*, *n* = 22; DATcre+;NR1*^flox/flox^*, *n* = 26; reflects data exclusion from 11 mice due to technical failures with the operant chambers, e.g., pellet dispenser or lever failures) were introduced to limited access to chow in their home cages in order to achieve body weights ∼85% of free-feeding levels. Mice were exposed to 0.5 g of the reinforcer pellets (14 mg Dustless Precision Pellets, used in subsequent behavioral experiments; BioServ) in their home cages during the first day of food restriction. Body weight was maintained at this level throughout the experiment, and standard chow was provided in the home cage at least 1 h after daily testing. Mice were trained on sequential days in extra wide aluminum and polycarbonate Med Associates modular mouse-testing chambers, each stationed inside a sound-attenuating chamber and equipped with a white noise generator, house light (both always on during all experiments), and a tone generator. A horizontal array of five illuminable nose-poke apertures formed one side of the box, and on the other resided an illuminable pellet-delivery magazine with an entry-detection photocell. Chambers also contained two retractable ultrasensitive mouse levers (2 g force requirement for actuation; Med Associates); these were positioned one each on both sides of the food magazine.

Training began with 2 d of familiarization to delivery of food pellets to the magazine. Fifty pellets were delivered to the magazine on a fixed-time 30 s schedule, each followed by a 2 s illumination of the magazine. Ten daily 30 min sessions of instrumental training followed. Sessions began with the extension of both levers, and responses on the active lever (designated left vs right in a counterbalanced fashion across genotypes) resulted in a 50 ms tone pulse, which was accompanied by pellet delivery and a 2 s illumination of the magazine light upon completion of the ratio schedule. The first 10 pellets per session were delivered on a fixed-ratio 1 schedule; subsequently, pellets were delivered on a variable-ratio 2 schedule. Responses to the inactive lever were recorded but had no programmed consequence. A 0.5 s timeout followed each pellet delivery, during which responses could not elicit delivery of another reward, but did count toward completion of the next reinforcement schedule.

### Sign tracking/goal tracking

**Figure 1:**
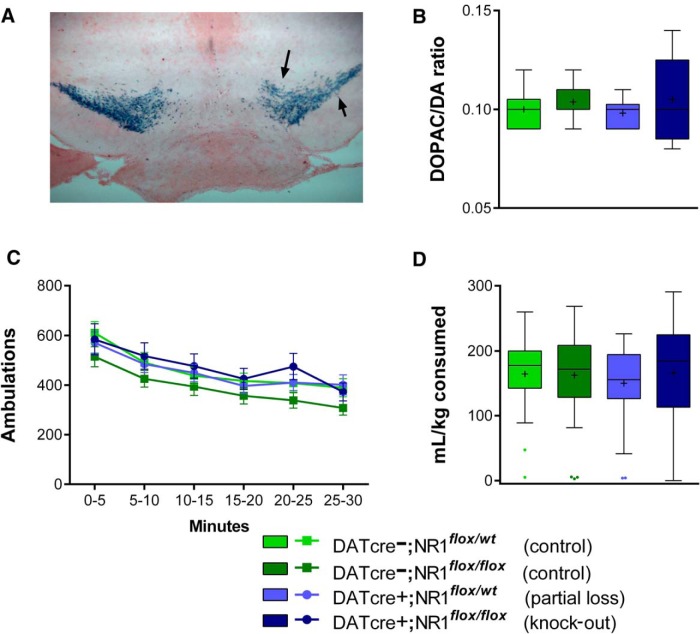
Initial characterization of the DATcre;NR1 mouse. ***A***, Prominent Cre-mediated recombination is seen in the midbrain of DATcre+ mice crossed with ROSA26-LacZ mice; arrows indicate ventral tegmental and substantia nigra pars compacta nuclei. ***B***, Ventral striatum dopamine turnover is indistinguishable among the four combinations of DATcre and NR1 genotypes. ***C***, No genotype effects were found over successive 5 min bins of locomotor behavior, and ***D***, levels of consumption of a 10% sweetened condensed milk solution were similar across all genotypes.

**Figure 2:**
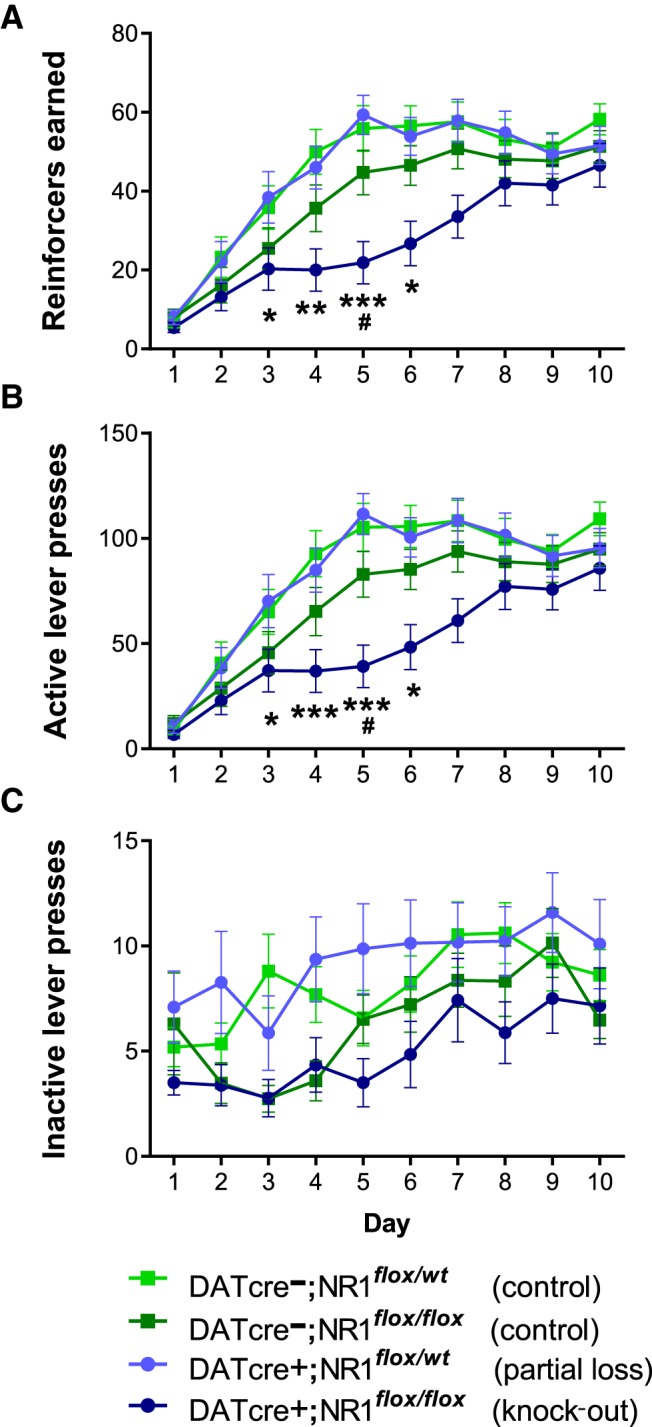
Loss of NMDA receptors in dopamine neurons impairs instrumental learning. ***A***, DATcre+;NR1*^flox/flox^* mice earn less reinforcers over 10 d of instrumental learning and make less active lever presses (***B***), but press the inactive lever at levels similar to the three other genotypes (***C***). *p < 0.05, **p < 0.01, ***p < 0.001 DATcre+;NR1***^flox/flox^*** versus DATcre+;NR1*^flox/wt^*; #*p* < 0.05 DATcre+;NR1*^flox/flox^* versus DATcre**—**;NR1*^flox/flox^* mice.

Methods for sign-tracking/goal-tracking pavlovian learning were modeled after Flagel et al. (2011). In the instrumental conditioning studies (above), DATcre+;NR1*^flox/wt^* mice were phenotypically similar to DATcre−;NR1*^flox/wt^* and DATcre—;NR1*^flox/flox^* control groups (Figures 1, 2), indicating that they could act as adequate controls; here, we treated them as such and compared their behavior with DATcre+;NR1*^flox/flox^* animals. A set of 63 experimentally naive animals was used (males only, DATcre+;NR1*^flox/wt^*, *n* = 32; DATcre+;NR1*^flox/flox^*, *n* = 31). We also tested DATcre—;NR1*^flox/wt^* animals (males, *n* = 31) to provide further empirical support for the validity of comparisons between DATcre+;NR1*^flox/wt^* and DATcre+;NR1*^flox/flox^* animals. The same schedule of caloric restriction described above was initiated prior to behavioral training. Animals first underwent 2 d of magazine training in which 30 food pellets were delivered to the magazine on a variable-time 60 s schedule. Fifteen daily sessions of sign-tracking/goal-tracking conditioning began the next day. These sessions consisted of 15 presentations on a variable-time 180 s schedule of a CS (“lever-CS”). Each lever-CS involved a 20 s extension of the lever to the right of the food magazine; two food pellets were delivered to the magazine coincident with lever-CS termination. Actuations of the lever-CS were recorded but had no programmed consequences.

On the day following the last conditioning session, all mice underwent a single test of conditioned reinforcement, wherein the two most lateral nose-poke apertures were illuminated. Responses to the active aperture (designated left vs right in a counterbalanced fashion across genotypes) resulted in a 5 s extension of the lever-CS, while responses to the inactive aperture were recorded but were without programmed effect. No food was delivered during this session. The session ended 60 min after the first active aperture response or after 90 min had elapsed, whichever occurred first.

### Data analysis

Statistical tests, outlined in Table 1, were conducted using Stata 13 (StataCorp LP). In all omnibus tests, DATcre (+ vs −) and NR1 (*flox/wt* vs *flox/flox*) zygosity were entered as between-subjects factors. In sign-tracking/goal-tracking experiments, for comparisons between DATcre+;NR1*^flox/wt^* and DATcre+;NR1*^flox/flox^* mice, NR1 genotype was the singular between-subjects factor; for comparisons between DATcre—;NR1*^flox/wt^* and DATcre+;NR1*^flox/wt^* mice, DATcre genotype was the singular between-subjects factor.

**Table 1:** Statistical tests used to analyze data

	**Data structure**	**Type of test**	***Post-hoc* power**
*a*	Normal	2 × 2 ANOVA	0.06
*b*	Negative binomial (overdispersed count)	GLMM, RI, and S	^a^
*c*	Normal	2 × 2 × 6 repeated-measures ANOVA	0.27
*d*	Negative binomial	GLMM, RI, and S with UCS matrix	^a^
*e*	Negative binomial	GLMM, RI, and S with UCS matrix (test of simple effects)	^a^
*f*	Negative binomial	GLMM, RI, and S with UCS matrix (Bonferroni-corrected *post-hoc* comparisons)	^a^
*g*	Negative binomial	GLMM, RI, and S with UCS matrix (test of simple effects)	^a^
*h*	Negative binomial	GLMM, RI, and S with UCS matrix (Bonferroni-corrected *post-hoc* comparisons)	^a^
*i*	Negative binomial	GLMM, RI, and S with UCS matrix (test of simple effects)	^a^
*j*	Negative binomial	GLMM, RI, and S with UCS matrix	^a^
*k*	Negative binomial	GLMM, RI, and S with UCS matrix (test of simple effects)	^a^
*l*	Negative binomial	GLMM, RI, and S with UCS matrix (Bonferroni-corrected *post-hoc* comparisons)	^a^
*m*	Negative binomial	GLMM, RI, and S with UCS matrix (Bonferroni-corrected *post-hoc* comparisons)	^a^
*n*	Negative binomial	GLMM, RI	^a^
*o*	Binomial	GLMM, RI, and S with UCS matrix	^a^
*p*	Negative binomial	GLMM, RI, and S with UCS matrix	^a^
*q*	Normal	GLMM, RI, and S	^a^
*r*	Normal	GLMM, RI, and S with UCS matrix	^a^
*s*	Normal	GLMM, RI, and S with UCS matrix (Bonferroni-corrected *post-hoc* comparisons)	^a^
*t*	Binomial	GLMM, RI	^a^
*u*	Negative binomial	GLMM, RI, and S	^a^
*v*	Log-transformed normal	GLMM, RI, and S	^a^
*w*	Binomial	GLMM, RI, and S with UCS matrix	^a^
*x*	Negative binomial	GLMM, RI, and S with UCS matrix	^a^
*y*	Normal	GLMM, RI, and S	^a^
*z*	Normal	GLMM, RI, and S with UCS matrix	^a^
*aa*	Binomial	GLMM, RI	^a^
*bb*	Binomial	GLMM, RI	^a^
*cc*	Negative binomial	GLMM, RI, and S	^a^
*dd*	Log-transformed normal	GLMM, RI, and S	^a^
*ee*	No assumptions made	Spearman’s ρ nonparametric correlation	>0.96^b^
*ff*	No assumptions made (underlying distributions unknown; high kurtosis and skew)	Wilcoxon rank sum nonparametric test (two-sample Mann–Whitney)	0.05, 0.07^c^
*gg*	Proportions	Fisher's exact test for cross-tabs	0.12
*hh*	Normal	Independent samples *t* tests, equal variances (tested by Levene’s test)	0.08, 0.08, and 0.09
*ii*	No assumptions made (goal-tracker distribution non-normal)	Wilcoxon rank sum nonparametric test (two-sample Mann–Whitney)	1.00^c^

GLMM, generalized linear mixed model; RI, random intercept; S, random slope (of repeated measure; UCS, unstructured covariance matrix between random effects (UCS matrix; covariance was fixed to zero in other GLMM models). Estimates of observed (*post hoc*) power are for experimentally relevant interaction effects.

a Estimates for main effects and interactions in GLMMS with RI and/or S, and for normally distributed data with RI and S are not readily calculable. This is the result of the complex, nonclosed form nature of optimizations of GLMMs with multiple random effects, which renders estimation of power not directly derivable, nor estimation via brute force, highly repeated simulation readily feasible.

b Simulation assumes normal distributions.

c Simulations assume (fitted) Weibull distributions.

All datasets were inspected for conformity to assumptions of the general linear model. Where assumptions were met, data were analyzed by univariate or repeated-measures ANOVA, with *t* tests where appropriate. For locomotor and learning experiments, we found significant departures from assumptions of traditional repeated-measures ANOVA, including violations of sphericity and/or heterogeneous, correlated residuals. These were not entirely unexpected, especially in our learning experiments, because correlations between testing days change as behavior progressively changes. Because population-level analysis often does not accurately characterize individual learning curves (Lashley, 1942; Estes, 1956; Gallistel et al., 2004; Verbeke and Molenberghs, 2009), generalized linear mixed models were used as a means to address these assumption violations, leading to better fits of the data by allowing subjects to vary with respect to intercepts and slopes and accommodating non-normal data distributions and nonconstant error variances/covariances. Models were fitted via maximum likelihood with cluster robust SEs using mean-variance adaptive Gauss–Hermite quadrature. Random subject-specific intercepts and/or linear slopes across days and their covariance were included on the basis of significantly improved model fit (tested via likelihood ratio testing of nested models). Distribution and link functions were chosen on the basis of properties of the variable studied and normality of the model residuals. Continuous data were analyzed using Gaussian identity-link models (i.e., linear mixed models); heavily skewed continuous data were modeled as log-normal. Log-link negative binomial models were applied to overdispersed count data and binomial logit models were applied to probability data. Statistics presented are tests of fixed effects. Wald $\chi^{2}$ tests of main effects and interactions were followed by contrasts of simple effects and, where appropriate, Bonferroni-adjusted tests of means.

Locomotor behavior measures (number of *x*-axis beam breaks) were analyzed across 5 min time bins; the bin was treated as a linear covariate. Free-food consumption (ml/kg consumed) was analyzed with day of measurement as a repeated measure. Because water consumption levels were negligible, these data were not analyzed. Dopamine utilization was analyzed as the ratio of metabolite DOPAC content to dopamine content.

In all learning experiments, training day was treated as continuous covariate, initially as a quadratic effect (i.e., curvilinear regression); if no quadratic effect of day was detected, it was removed, leaving the linear effect. For instrumental learning, reinforcers earned across days were analyzed, as were active and inactive lever presses. For sign-tracking/goal-tracking data, we analyzed genotype effects on behavioral data acquired across successive sessions, mirroring the analysis in Flagel et al. (2011). Sign tracking was quantified by analyzing (1) the probability of lever contact (contacts were defined as full actuations of the lever-CS) during lever-CS presentation, (2) total number of lever contact responses, and (3) latency to contact the lever. Goal tracking was similarly measured as the (1) probability of making a head entry into the magazine during a lever-CS presentation, (2) total number of head entries during the lever-CS presentations, and (3) latency to enter the magazine upon lever-CS presentation. A “conditioning ratio” measure of discriminative responding was also formed from goal-tracking data, calculated by comparing magazine head entries during the CS to those made during a time period of equivalent duration immediately preceding the CS (the latter termed the pre-CS period): $\frac{\text{CS magazine entries}}{\text{CS entries}+\text{ Pre-CS entries}}$.

We also calculated proposed conditional response “bias” measures described by Meyer et al. (2012), wherein phenotypic tendency toward sign tracking versus goal tracking is quantified by the following: (1) differences in response probabilities, Pr(lever contact) − Pr(magazine entry), (2) a discrimination index of responses, $\frac{\text{lever contacts}-\text{magazine entries}}{\text{lever contacts}+\text{magazine entries}}$, and (3) relative response latencies, $\frac{\bar{x}\text{ magazine entry latency}-\bar{x}\text{ lever contact latency}}{\text{CS duration (20 sec)}}$. These three indices ranged from +1 to −1, representative of bias toward sign tracking versus goal tracking, respectively. Their correlational structure was explored, and they were then averaged to form a conditional approach “summary bias score.” Summary bias scores were further averaged over three session blocks. Distributions of summary scores at the start and end of training were analyzed using nonparametric tests. To investigate whether any genotype effects on sign tracking were obscured by analysis of all subjects’ behavior simultaneously, we used the final summary bias score (from the last three sessions) to designate mice as either a sign tracker or goal tracker on the basis of whether their score was positive or negative, respectively. Genotype effects on designation distribution were analyzed with Fisher’s exact test. We then plotted sign-trackers’ behavior and goal-trackers’ behavior separately, visualizing learning rates within each genotype/conditional response type combination.

Data from six subjects on day 8 were lost due to technical failure. These data points were treated as missing at random in mixed model analysis.

Measures of responding for conditioned reinforcement included number of lever-CSs earned and number of active and inactive aperture nose pokes. Because sign tracking has been associated with greater conditioned reinforcement in rats (Robinson and Flagel, 2009; Flagel et al., 2011; Lomanowska et al., 2011), we also compared number of lever-CSs earned by animals designated sign trackers with numbers earned by animals designated goal trackers to establish whether the same relationship exists in mice.

Figures are presented as mean ± SE line plots or as Tukey box-plots, the latter demonstrating spread about a group median with plus symbols (+) demarcating group means.

## Results

### Baseline characterization

In Figure 1*A*, Cre-mediated gene recombination can be seen prominently in the substantia nigra pars compacta and the ventral tegmental area of the DATcre+ mouse, consistent with its initial characterization (Bäckman et al., 2006). Quantification of monoamine utilization by HPLC indicated that neither the DATcre construct, nor the floxed NR1 gene or its excision in DATcre+ subjects, affected basal dopamine utilization within the ventral striatum (Figure 1*B*; DATcre: *F*_(1,31)_ = 0.01, *p* = 0.932; NR1: *F*_(1,31)_ = 1.51, *p* = 0.229; DATcre x NR1: *F*_(1,31)_ = 0.14, *p* = 0.713, *a* in Table 1).

DATcre–;NR1*^flox/wt^*, DATcre–;NR1*^flox/flox^*, DATcre+;NR1*^flox/wt^*, and DATcre+;NR1*^flox/flox^* mice were initially characterized for total ambulatory activity in a novel environment. As depicted in Figure 1*C*, mice exhibited reduced locomotor behavior over time as they habituated to their surroundings; however, no main effects or interactions involving DATcre genotype or NR1 genotype were detected (DATcre: Wald $\chi_{\left(1\right)}^{2}$ = 0.12, *p* = 0.731; NR1: Wald $\chi_{\left(1\right)}^{2}$ = 1.88, *p* = 0.171; DATcre × NR1: Wald $\chi_{\left(1\right)}^{2}$ = 0.51, *p* = 0.474; DATcre × NR1 × time bin: Wald $\chi_{\left(1\right)}^{2}$ = 0.03, *p* = 0.863, *b*), indicating that that locomotor behavior was unaffected by genetic manipulation of NMDAR in dopamine cells. All mice increased consumption of the sweetened condensed milk solution across successive days of access (Day: *F*_(1,320)_ = 12.38, *p* = 0.0005), but as depicted in Figure 1*D*, no effects of genotype were detected (DATcre: *F*_(1,320)_ = 0.06, *p* = 0.804; NR1: *F*_(1,320)_= 0.02, *p* = 0.902; DATcre × NR1: *F*_(1,320)_= 0.64, *p* = 0.423; DATcre × NR1 × day: *F*_(1,320)_ = 0.001, *p* = 0.962, *c*).

### Instrumental learning

Reinforcers earned during the instrumental conditioning sessions are depicted in Figure *2A*. Here, mixed model revealed significant DATcre × day (Wald $\chi_{1}^{2}$= 4.24, *p* = 0.039), NR1 × day (Wald $\chi_{\left(1\right)}^{2}$ = 4.65, *p =* 0.031), and DATcre x NR1 × day interactions (Wald $\chi_{\left(1\right)}^{2}\;$= 7.38, *p* = 0.007, *d*). The NR1 × day interaction was significant within DATcre+ animals (within DATcre+, Wald $\chi_{\left(1\right)}^{2}$ = 15.55, *p* = 0.001; within DATcre–, Wald $\chi_{\left(1\right)}^{2}$ = 0.18, *p* = 0.673, *e*), and successive Bonferroni-corrected contrasts revealed that while behavior during the initial training sessions did not differ, DATcre+;NR1*^flox/flox^* mice earned fewer reinforcers than DATcre+;NR1*^flox/wt^* mice on days 3–6 (Day 3: Wald $\chi_{\left(1\right)}^{2}$ = 9.01, *p* = 0.027; Day 4: Wald $\chi_{\left(1\right)}^{2}$ = 13.69, *p* = 0.002; Day 5: Wald $\chi_{\left(1\right)}^{2}$ = 16.89, *p* < 0.001; Day 6: Wald $\chi_{\left(1\right)}^{2}$ = 9.49, *p* = 0.021, *f*). Similar findings were obtained when the omnibus interaction was explored via simple effects within NR1 genotypes (within NR1*^flox/flox^*, DATcre × day: Wald $\chi_{\left(1\right)}^{2}$ = 11.93, *p* = 0.0006; within NR1*^flox/wt^*, DATcre × day: Wald $\chi_{\left(1\right)}^{2}$ = 0.25, *p* = 0.620, *g*). DATcre+;NR1*^flox/flox^* earned fewer reinforcers than DATcre—;NR1*^flox/flox^* mice on day 5 (Wald $\chi_{\left(1\right)}^{2}$ = 10.14, *p* = 0.014), and a similar trend was found on day 6 (Wald $\chi_{\left(1\right)}^{2}$ = 7.41, *p* = 0.065, *h*). Importantly, no differences in instrumental behavior between DATcre–;NR1*^flox/flox^*, DATcre–;NR1*^flox/wt^*, and DATcre+;NR1*^flox/wt^* mice were detected (genotype: Wald $\chi_{\left(2\right)}^{2}$ = 4.07, *p =* 0.133; genotype × day: Wald $\chi_{\left(2\right)}^{2}$ = 0.76, *p =* 0.683, *i*).

To provide evidence that this difference in instrumental responding reflected differences in associative behavior, similar analyses were performed on number of active (reinforced) lever (Figure 2*B*) and inactive (nonreinforced) lever (Figure 2*C*) presses. A DATcre × NR1 × day interaction for active lever presses (Wald $\chi_{\left(1\right)}^{2}\;$= 8.27, *p* = 0.004, *j*) was decomposed (within DATcre+, Wald $\chi_{\left(1\right)}^{2}$ = 18.21, *p* = 0.00001; within DATcre–, Wald $\chi_{\left(1\right)}^{2}$ = 0.19, *p* = 0.664, *k*) to reveal that fewer active lever presses were made by DATcre+;NR1*^flox/flox^* mice relative to DATcre+;NR1*^flox/wt^* mice, again on days 3–6 (Day 3: Wald $\chi_{\left(1\right)}^{2}$ = 9.36, *p* = 0.022; Day 4: $\chi_{\left(1\right)}^{2}$ = 14.88, *p* = 0.001; Day 5: $\chi_{\left(1\right)}^{2}$ = 18.45, *p* = 0.0002; Day 6: $\chi_{\left(1\right)}^{2}$ = 9.72, *p* = 0.018, *l*). Fewer active lever presses were also made by DATcre+;NR1*^flox/flox^* mice relative to DATcre+;NR1*^flox/wt^* on day 5 (Wald $\chi_{\left(1\right)}^{2}$ = 11.55, *p* = 0.007), with near-significant differences on day 6 (Wald $\chi_{\left(1\right)}^{2}$ = 7.72, *p* = 0.054, *m*). On the other hand, no interactions with genotypes were found for inactive lever pressing (DATcre × NR1: Wald $\chi_{\left(1\right)}^{2}$ = 0.35, *p* = 0.065; DATcre x NR1 × day: Wald $\chi_{\left(1\right)}^{2}$ = 0.60, *p* = 0.439, *n*), indicating that the impairment in instrumental behavior observed in animals lacking NMDA receptors in dopamine neurons was selective to the active lever.

### Sign tracking/goal tracking

**Figure 3:**
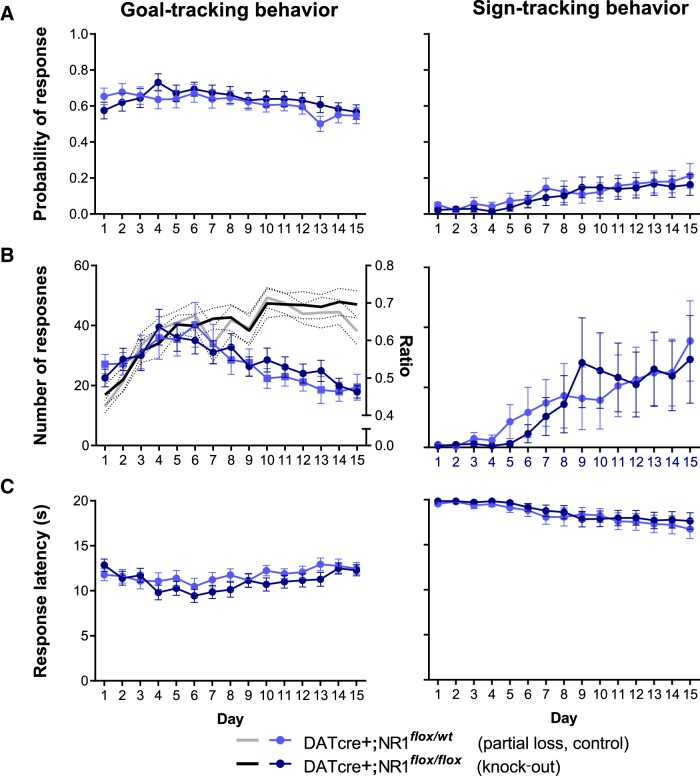
Genetic deletion of NMDAR in dopamine neurons is without effect on sign-tracking or goal-tracking responses during pavlovian approach learning. Mice with two floxed NR1 alleles (knock-outs) engage in goal-tracking and sign-tracking behaviors at levels similar to heterozygote controls, as measured by probability of a single magazine entry (left) or lever contact (right) during lever-CS presentation (***A***) and number of magazine head entries (left) and lever contacts (right) during lever-CS presentation (***B***). For head entries (left), the ratio between responding during the CS and pre-CS (the latter an equivalent duration preceding period; see Materials and Methods), a measure of discriminative approach behavior, is plotted on the *y*-axis (right). Genotype also did not affect latency to enter the magazine (left) or contact the lever-CS (right) upon its extension (***C***).

The acquisition of both sign-tracking and goal-tracking conditional responses is depicted in Figure 3, using the dependent measures described in Flagel et al. (2011). Because we present quantitative measures of both goal tracking and sign tracking from the same subjects (rather than segregating subjects as expressing one response or the other; see Figure 5), the slope of goal-tracking learning curves appears modest; discrimination ratios, however, indicate clear evidence of learning. Goal tracking tended to be expressed first (likely due to the fact that we conducted magazine training prior to pavlovian conditioning), as can occur in rats (Meyer et al., 2012). In a subset of animals, goal-tracking is then diminished as it undergoes response competition during the emergence of sign-tracking behaviors. Importantly, in this subpopulation, we detected both reliable and vigorous sign-tracking behavior.

Analyses comparing goal-tracking behavior (Figure 3*A–C*, left) of DATcre+;NR1*^flox/flox^* mice and DATcre+;NR1*^flox/wt^* mice revealed no effects of genotype on the probability of making a magazine head entry during the lever-CS (genotype: Wald $\chi_{\left(1\right)}^{2}$ = 0.29, *p* = 0.592; genotype × day: Wald $\chi_{\left(1\right)}^{2}$ = 1.22, *p* = 0.268, *o*); moreover, there were no effects of genotype on number of magazine entries during the lever-CS (genotype: Wald $\chi_{\left(1\right)}^{2}$ = 0.74, *p* = 0.389; genotype × day: Wald $\chi_{\left(1\right)}^{2}$ = 0.66, *p* = 0.418, *p*). We also analyzed the discrimination ratio between CS and pre-CS period responding, again finding no genotype effects (plotted on right-hand *y*-axis of Figure 3*B*; genotype: Wald $\chi_{\left(1\right)}^{2}$ = 0.01, *p* = 0.913; genotype × day: Wald $\chi_{\left(1\right)}^{2}$ = 0.76, *p* = 0.384, *q*). Though a significant day × genotype effect on latency to enter the magazine upon lever-CS onset was found (Wald $\chi_{\left(1\right)}^{2}$ = 3.90, *p* = 0.048, *r*), *post hoc* tests did not reveal any significant differences between groups on any of the 15 d of training (all *p*s > 0.076 uncorrected for multiple comparisons; all *p*s = 1.000 Bonferroni corrected, *s*). Thus, with the exception of a marginal omnibus test suggesting deviations in response latency, these analyses do not support altered discriminated goal approach after loss of NMDA receptors in dopamine neurons.

Surprisingly, analyses of the development of corresponding lever-CS approach/sign-tracking behaviors (Figure 3*A–C*, right) also did not reveal evidence of genotype effects: DATcre+;NR1*^flox/flox^* mice and DATcre+;NR1*^flox/wt^* mice approached and actuated with the lever-CS with similar probabilities as conditioning sessions progressed (genotype: Wald $\chi_{\left(1\right)}^{2}$ = 1.48, *p* = 0.223; genotype × day: Wald $\chi_{\left(1\right)}^{2}$ = 0.001, *p* = 0.969, *t*), making similar numbers of lever contacts (genotype: Wald $\chi_{\left(1\right)}^{2}$ = 1.49, *p* = 0.222; genotype × day: Wald $\chi_{\left(1\right)}^{2}$ = 1.01, *p* = 0.314, *u*) and doing so with latencies that did not differ (genotype: Wald $\chi_{\left(1\right)}^{2}$ = 0.35, *p* = 0.553; genotype × day: Wald $\chi_{\left(1\right)}^{2}$ = 0.03, *p* = 0.874, *v*).

We also compared NR1*^flox/wt^* animals that were either DATcre+ or DATcre— to establish whether Cre-mediated deletion of a single NR1 allele was sufficient to alter goal-tracking or sign-tracking responses. Analyses of these two groups indicated that goal-tracking behaviors were not significantly different (probability of head entry during lever-CS, genotype: Wald $\chi_{\left(1\right)}^{2}$ = 0.04, *p* = 0.839; genotype × day: Wald $\chi_{\left(1\right)}^{2}$ = 0.49 *p* = 0.484, *w*; number of head entries, genotype: Wald $\chi_{\left(1\right)}^{2}$ = 0.04, *p* = 0.850; genotype × day: Wald $\chi_{\left(1\right)}^{2}$ = 0.25 *p* = 0.616, *x*; discrimination ratio, genotype: Wald $\chi_{\left(1\right)}^{2}$ = 0.73, *p* = 0.393, genotype × day: Wald $\chi_{\left(1\right)}^{2}$ = 0.04 *p* = 0.842, *y*; magazine entry latency, genotype: Wald $\chi_{\left(1\right)}^{2}$ = 0.07, *p* = 0.784; genotype × day: Wald $\chi_{\left(1\right)}^{2}$ = 0.25 *p* = 0.833, *z*). DATcre—;NR1*^flox/wt^* and DATcre+;NR1*^flox/wt^* differed in their probability of actuating the lever during a lever-CS (genotype: Wald $\chi_{\left(1\right)}^{2}$ = 5.91, *p* = 0.015; genotype × day: Wald $\chi_{\left(1\right)}^{2}$ = 3.77, *p* = 0.052, *aa*). Nevertheless, like DATcre+;NR1*^flox/wt^* mice, when DATcre—;NR1*^flox/wt^* animals were compared with DATcre+;NR1*^flox/flox^* knock-out mice lacking both NR1 alleles in dopamine neurons, no differences were found (genotype: Wald $\chi_{\left(1\right)}^{2}$ = 0.10, *p* = 0.755; genotype × day: Wald $\chi_{\left(1\right)}^{2}$ = 1.83, *p* = 0.176, *bb*). DATcre—;NR1*^flox/wt^* and DATcre+;NR1*^flox/wt^* expressed other sign-tracking measures at similar rates (number of lever contacts, genotype: Wald $\chi_{\left(1\right)}^{2}$ = 1.67, *p* = 0.196; genotype × day: Wald $\chi_{\left(1\right)}^{2}$ = 0.26, *p* = 0.610, *cc*; lever contact latency, genotype: Wald $\chi_{\left(1\right)}^{2}$ = 0.23, *p* = 0.629; genotype × day: Wald $\chi_{\left(1\right)}^{2}$ = 0.08 *p* = 0.778, *dd*). Thus, the behavior DATcre—;NR1*^flox/wt^* and DATcre+;NR1*^flox/wt^* was generally equivalent, supporting the use of DATcre+;NR1*^flox/wt^* as controls with DATcre+;NR1*^flox/flox^* knock-outs for the main comparisons described above.

**Figure 4:**
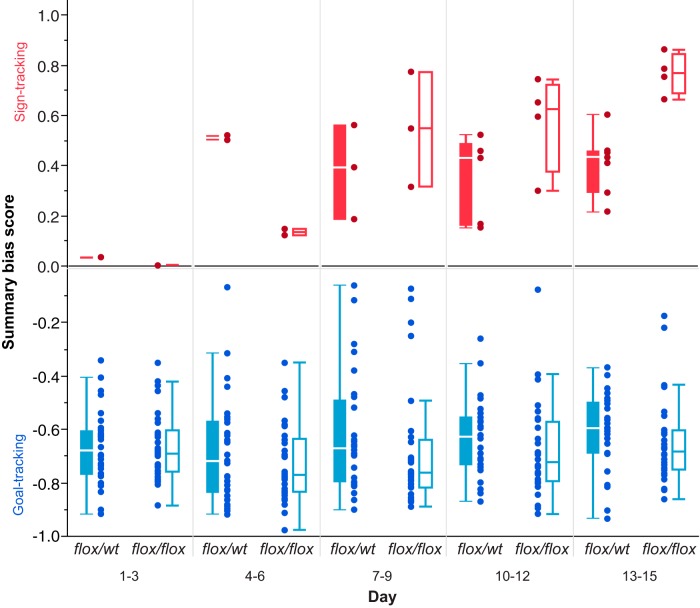
Distributions of conditional approach summary bias. Summary bias scores, formed from relative probability, response, and latency data for sign-tracking and goal-tracking responses for individual mice (see Materials and Methods), are plotted in 3 training day bins. Positive values indicate a tendency to sign track and negative values indicate a tendency to goal track. Goal tracking is dominant early in training, but sign tracking emerges progressively across successive days; however, no significant differences in score distributions were found between genotypes. Closed box-plots, DATcre+;NR1*^flox/wt^* (partial loss control, “flox/wt”); open box-plots, DATcre+;NR1*^flox/flox^* (knock-out, “flox/flox”).

Because sign-tracking responses tend to come at the expense of goal-tracking responses, and vice versa, we calculated relative response bias scores on the basis of probabilities, responses, and latencies to respond to the lever-CS versus the food magazine (see Materials and Methods). Individual scores, averaged across 3 d blocks, demonstrated significant pairwise correlations that increased in magnitude across training (Days 1–3: probability vs response, Spearman’s ρ = 0.367, *p* = 0.003; probability vs latency, Spearman’s ρ = 0.934, *p* < 0.001; latency vs response, Spearman’s ρ = 0.328, *p* = 0.009; Days 13–15: probability vs response, Spearman’s ρ = 0.720, *p* < 0.001; probability vs latency, Spearman’s ρ = 0.969, *p* < 0.001; latency vs response, Spearman’s ρ = 0.698, *p <* 0.001, *ee*). The three scores were then averaged to form a summary bias score, as described previously (Meyer et al., 2012). Plotted in Figure 4, summary bias scores above zero indicate a tendency to sign track rather than goal track, and negative values correspond to a bias toward goal tracking. At the start of training, summary bias scores were similar in both genotypes (Wilcoxon rank sum, Days 1–3: z = 0.007, *p =* 0.994), and no genotype differences were found by the conclusion of testing (Wilcoxon rank sum, Days 13–15, z = 1.650, *p =* 0.099, *ff*).

**Figure 5:**
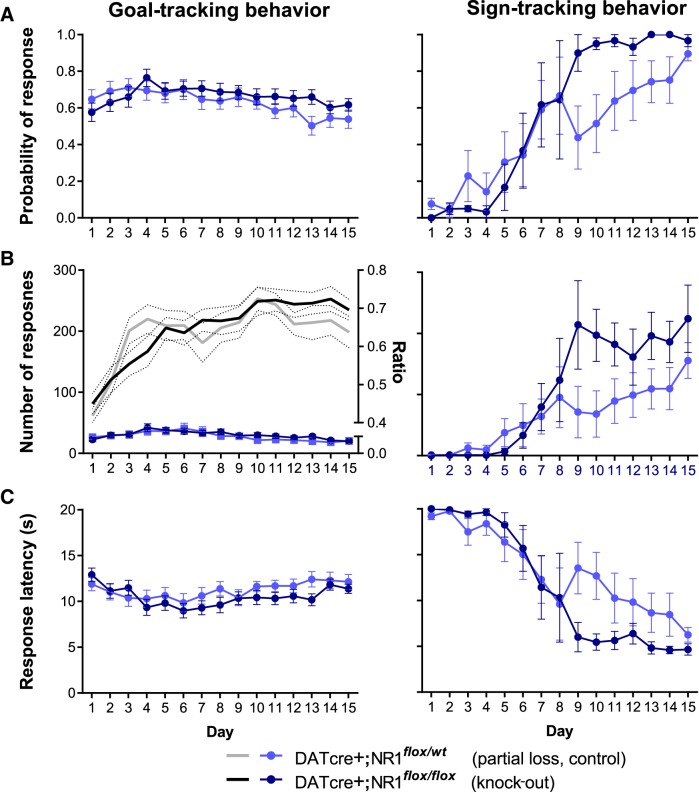
Behavior plotted according to conditional response designation. Animals with a positive summary bias score for days 13–15 were designated sign trackers; those with negative scores were designated sign trackers. As in Figure 3, probability of a single magazine entry (left) or lever contact (right) during lever-CS presentation (***A***); number of magazine head entries (left) and lever contacts (right) during lever-CS presentation, with the ratio between CS and pre-CS responding plotted for head entries on the right-hand *y*-axis (***B***); and latency to enter the magazine (left) or contact the lever-CS (right) upon its extension (***C***) are measured. Sign-tracking DATcre+;NR1***^flox/flox^***mice appear to display a greater degree of sign-tracking behaviors than controls.

We designated mice as sign trackers or goal trackers according to whether their final 3 d average summary bias scores were positive or negative. A bias toward the sign-tracking conditional response, under this scheme of categorization, occurred in fewer mice than did goal tracking (*n* = 11 vs *n* = 52; *n* = 5 additional mice were found to make sign-tracking responses, but in magnitudes that did not exceed their goal-tracking behaviors). Relative rates of phenotype designations did not differ between DATcre+;NR1*^flox/flox^* mice and DATcre+;NR1*^flox/wt^* mice (7 of 31 and 4 of 32, respectively, designated sign trackers; Fisher’s exact test, *p =* 0.337, *gg*). Reasoning we might be able to more sensitively observe differences in the rate of learning by examining only their respective conditional response, we assessed the acquisition of goal-tracking behaviors in goal trackers exclusively and the acquisition of sign-tracking behaviors in sign trackers exclusively, as shown in Figure 5*A–C* (left and right, respectively). Though goal-tracking behavior appears similar to that expressed by the sample as a whole, visual inspection of Figure 5 suggests that among sign trackers, DATcre+;NR1*^flox/flox^* mice express a greater degree of sign-tracking behavior–opposite of the hypothesized effect. However, because comparisons of summary bias score distributions did not reach traditional levels of statistical significance, no additional exploratory statistical evaluations of these data were conducted.

**Figure 6:**
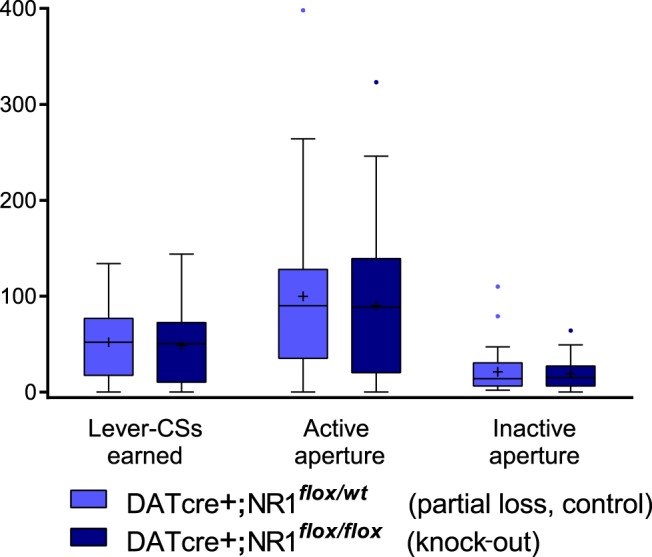
Test of conditioned reinforcement. Mice were allowed to earn brief presentations of the lever-CS by performing a novel instrumental response. No differences between genotypes in the number of lever-CSs earned, and nor responses to the active or inactive nose-poke apertures were found.

Finally, the ability of the lever-CS to support new learning via conditioned reinforcement, a phenomenon elevated in sign-tracking animals (Robinson and Flagel, 2009; Flagel et al., 2011; Lomanowska et al., 2011) and considered reflective of incentive motivational properties acquired by cues (Berridge, 2000; Flagel et al., 2009), was evaluated. Mice were allowed to make nose-poke responses to elicit brief presentations of the lever-CS during a single session that followed the last day of conditioning. As shown in Figure 6, the number of lever-CS presentations earned by DATcre+;NR1*^flox/wt^* mice and DATcre+;NR1*^flox/flox^* mice did not significantly differ (*t*_(61)_ = 0.526, *p* = 0.601), nor did the number of active aperture (*t*_(61)_ = 0.559, *p* = 0.551) or inactive aperture (*t*_(61)_ = 0.553, *p* = 0.581, *hh*) responses. However, as occurs in rats, mice designated sign trackers exhibited higher levels of conditioned reinforcement than mice designated goal trackers, earning more lever-CS presentations (Wilcoxon rank sum, z = −2.608, *p* = 0.009, *ii*).

## Discussion

Here, the effects of genetic excision of the NMDAR from dopamine neurons on associative reward-related learning were evaluated. Mice lacking NMDAR in dopamine neurons exhibited impaired instrumental learning but normal sign-tracking and goal-tracking responses in a pavlovian conditioning procedure. These results are presented against the backdrop of normal exploratory locomotion and palatable food consumption, eliminating these ancillary phenotypes as likely explanations for the observed learning effects.

### NMDAR activity in dopamine neurons contributes to acquisition of an appetitive instrumental response

Loss of NMDAR in dopamine neurons resulted in slower acquisition of instrumental responding; this finding is in general agreement with results gathered earlier using a similar mouse model (Zweifel et al., 2009). Qualitative aspects of the particular pattern of results offer indications of the nature of the behavioral deficit observed: the absence of group differences during the first and final days of training suggests that genotype did not affect baseline lever-pressing rates per se, and the similar asymptotic rates of pressing at the end of training indicates that motivation to obtain the food reward may not be sensitive to genotype. The impairments in a spontaneously acquired instrumental response were observed only during intermediate stages of the learning process, suggesting that phenotypic differences in mice lacking NMDAR in dopamine neurons relate to altered learning capabilities.

This result suggests a causal role for NMDA-mediated neurotransmission in dopamine neurons in instrumental learning. One possibility is that the loss of NMDA receptors disables one mechanism that contributes to phasic, stimulus-related dopamine neuron firing (Suaud-Chagny et al., 1992; Zweifel et al., 2009; Parker et al., 2010). Additionally, loss of the NMDAR eliminates NMDAR-mediated synaptic plasticity within dopamine neurons (Engblom et al., 2008; Zweifel et al., 2008; Luo et al., 2010). Thus, while the behavioral effect observed may well relate to altered phasic release, it is possible that other mechanisms are at play, including loss of synaptic plasticity between glutamatergic inputs and dopamine neurons or other downstream molecular changes. Because we did not measure NMDA expression or monitor dopamine activity in the context of behavior, it is difficult to disentangle these different interpretations, and further experiments are needed to parse these possibilities.

Our data are, however, consistent with a number of optogenetic studies wherein response-contingent optical activation of dopamine neurons either facilitated an appetitive instrumental response or was sufficient to support responding alone (Adamantidis et al., 2011; Witten et al., 2011; Kim et al., 2012). Moreover, after asymptotic acquisition of the relationship between a CS that was predictive of periods of response-contingent reward availability (i.e., a discriminative stimulus), transient optical activation of dopamine neurons delivered concurrently with presentation of a compound CS prevented the normally observed blocking effect (Steinberg et al., 2013). Further, the behavioral impact of unexpected negative shifts in outcome value was also diminished by activation of dopamine neurons. These findings are consistent with phasic dopamine acting as a prediction error signal that causally drives learning. The optogenetic studies are convincing, and thus we argue that the current data are parsimonious with a hypothesized role for phasic dopamine activity in reward learning. That said, it remains unclear how light-evoked events interact with ongoing endogenous phasic events and tonic activity states, and whether they reproduce the postsynaptic effects of normal stimulus-elicited phasic events. Moreover, experimenter-prescribed stimulation timing is likely unable to precisely mimic ongoing changes in temporal relationships between the onset of phasic dopamine bursts and environmental events, for example, as a stimulus-outcome relationship is learned and phasic signals shift from the time of reward delivery to cue onset (Ljungberg et al., 1992; Day et al., 2007). Here, we demonstrate that instrumental learning is modulated by NMDA-mediated activity in dopamine neurons and by putative attenuation of phasic dopamine signals that were endogenously generated by afferent inputs to dopamine neurons in response to environmental stimuli.

### Loss of NMDARs in dopamine neurons does not impact frequency or acquisition of a sign-tracker conditional response

Sign-tracking rats—those that approach and interact with a predictive cue (e.g., the extension of a lever-CS) during an autoshaping task, often at the expense of approaching the location of reward delivery (Williams and Williams, 1969)—are thought of as exhibiting a form of incentive salience attribution over and above the pavlovian contingency learning exhibited by goal-tracking rats. These differential conditional responses offer an opportunity to distinguish between the prediction error and incentive salience attribution perspectives of dopamine. Flagel et al. (2011) provided causal evidence that dopamine receptor activity is required for sign tracking, observing a deficit in acquisition of sign tracking, but not goal tracking, after treatment with a dopamine receptor antagonist. Importantly, sign-tracking animals also display more prominent CS-evoked dopamine as they learn their conditional response than do goal trackers (Flagel et al., 2011). Because goal trackers and sign trackers must both learn the contingency between the CS and the US to express their responses, it has been suggested that the phasic dopamine release patterns do not simply teach contingency learning. Phasic dopamine release is argued, in this case, instead to be necessary for a cue to acquire incentive properties, progressively increasing its motivational pull on behavior (and, correspondingly, progressively increasing sign-tracking conditional responses) as CS-US pairings continue (Flagel et al., 2011).

Because NMDAR loss in dopamine neurons results in attenuation of the magnitude of phasic dopamine release to ∼30% that of controls (Zweifel et al., 2009; Parker et al., 2010), this genetic model applied to the sign-tracker/goal-tracker paradigm offered an experimental design equipped to distinguish between prediction error and incentive salience perspective. If the relationship between the magnitude of CS-evoked dopamine and sign tracking is causal, we hypothesized that a putative reduction in the amplitude of NMDA-mediated dopamine release should reduce the frequency of sign-tracking behavior or the rate of its acquisition. We found no evidence to support this conclusion: for all dependent measures; no differences in the form of conditional responses expressed by mice lacking NMDAR in dopamine neurons and control mice were detected.

In addition to failing to support the incentive salience perspective of dopamine activity in reward learning, we also did not yield evidence of a contribution of NMDA-mediated dopamine activity and/or phasic release to pavlovian goal approach, nor have several others using a similar mouse genetics approach (Parker et al., 2010, 2011). Given that prediction error signals in the mesencephalon have been observed during pavlovian conditioning, across a wide variety of task conditions and parameters, but most extensively characterized within the context of appetitive pavlovian conditioning (Ljungberg et al., 1992; Schultz et al., 1993, 1997; Waelti et al., 2001; Fiorillo et al., 2003), and because pharmacological strategies have shown that the pavlovian approach has been shown to be dependent upon NMDAR activity in the ventral tegmental area (Stuber et al., 2008; Ranaldi et al., 2011), this is a surprising result.

One possibility is that the reported residual 30% phasic signal in the DATcre;NR1 mouse may be sufficient to support pavlovian approach learning. Given that reward preference and reward learning is possible even after massive dopamine depletions (Cannon and Palmiter, 2003; Robinson et al., 2005), this residual phasic activity may indeed provide more than adequate signal to noise necessary for pavlovian delay conditioning, especially when associative contingencies are binary and deterministic (i.e., P(US|CS) = 1, P(US|∼CS) = 0). The magnitude of midbrain neuron burst responses encodes the relative value of predictive stimuli (Fiorillo et al., 2003); perhaps a behavioral impairment would be revealed in a scenario where 30% of the normal signal-to-noise in dopamine neurons provides insufficient dynamic range (e.g., discriminating between two stimuli with marginal differences in predictive value). Given, however, that sign-tracker rats are distinguished from goal-tracking rats by a quantitative difference in CS-evoked dopamine (Flagel et al., 2011), if this difference causally influenced the form of conditional response expressed, we would still expect a measureable difference in the degree of sign-tracking behavior in DATcre+;NR1*^flox/flox^* mice that have a dramatic, albeit not full, diminution of phasic dopamine release. There was no indication of this in our data. Alternatively, it is possible that a loss of NMDAR-mediated synaptic plasticity or other NMDAR-dependent physiological mechanisms in mice lacking NMDAR in dopamine neurons obscured the observation of a behavioral difference in sign tracking. Additionally, it is possible that these behaviors may be supported by dopaminergic projections to the basolateral amygdala or prefrontal cortex, as these cells express very little DAT (Lammel et al., 2008); therefore, NR1 recombination may have not fully occurred. However, a study using PCR to detect recombination of NR1 in the SN/VTA in the same mouse model found successful recombination of NR1 in 34 of 36 such cells (Luo et al., 2010). Thus, Cre recombinase expression appears to be sufficient to drive excision of NR1 in the majority of dopaminergic neuronal populations, even those expressing very low levels of DAT.

### The role of NMDAR in dopamine neurons in reward-related behaviors

What is clear from these experiments is that NMDAR-mediated activity in dopamine neurons is not required to adaptively respond in the pavlovian approach paradigm. Interestingly, in addition to the pavlovian approach, other phenotypes that were historically thought to require NMDAR in dopamine neurons, such as sensitization to psychostimulants (Kalivas and Alesdatter, 1993; Wolf et al., 1994, 1998; Vanderschuren and Kalivas, 2000), have also turned out to be unaffected in their absence (Zweifel et al., 2008; Luo et al., 2010; Beutler et al., 2011). Given that the degree of NMDAR-dependent plasticity in dopamine neurons—expressed as increased AMPA receptor expression or current—induced by drugs of abuse correlates with degree of behavioral sensitization observed (Ungless et al., 2001; Borgland et al., 2004) and that NMDAR-dependent plasticity is observed selectively during periods of active learning of pavlovian conditioning (Stuber et al., 2008), these results are especially unanticipated. However, several studies have implicated NMDAR in non-dopaminergic cell types or brain regions as responsible for these phenomena (Luo et al., 2010; Beutler et al., 2011; Parker et al., 2011).

Because they co-occur and share dopaminergic substrates, locomotor sensitization to psychostimulants has been linked with heightened or sensitized incentive salience attribution (Robinson and Berridge, 1993; Wyvell and Berridge, 2001; Tindell et al., 2005; Olausson et al., 2006; Ostlund et al., 2014), including sign-tracking behavior (Doremus-Fitzwater and Spear, 2011). Supporting this link, we observed that elimination of NMDA receptors on dopamine neurons did not affect pavlovian sign tracking, and previous studies using similar models have also found locomotor sensitization is not dependent upon NMDARs in dopamine neurons (Engblom et al., 2008; Zweifel et al., 2008; Luo et al., 2010; Beutler et al., 2011). Thus, while a host of neural mechanisms likely influence the development of a sign-tracking conditional response (Flagel et al., 2007, 2010; Lomanowska et al., 2011; Fitzpatrick et al., 2013; Perez-Sepulveda et al., 2013; Haight and Flagel, 2014), our data indicate that NMDAR activity in dopamine neurons—along with its contribution to phasic dopamine release—is not among these factors.

Previous work has demonstrated persistent elevations in synaptic AMPA/NMDA ratios in dopamine neurons of animals self-administering cocaine, while AMPA/NMDA ratios were only transiently elevated in animals responding for food (Chen et al., 2008). NMDAR dynamics are therefore susceptible to modulation by rewarding experiences and reward modality. Thus, our observation that the acquisition and performance of pavlovian conditional responses were not different in mice lacking NMDAR in dopamine neurons may depend on the specific experimental conditions used here. We note, however, that enhanced AMPA/NMDA ratios have been observed during pavlovian conditioning for food (Stuber et al., 2008), and in a similar mouse model of loss of NMDARs in dopamine neurons, cue-based learning was impaired (Zweifel et al., 2009). Conversely, the acquisition of a pavlovian conditioned place preference for cocaine is unaffected in knock-out mice (Engblom et al., 2008; Luo et al., 2010; but see Zweifel et al., 2008). These data do not support the simple idea that NMDAR are involved in pavlovian responses to drugs but not food.

### Sign tracking in mice

Sign-tracking behavior comparable to that observed toward a lever-CS in rats has been difficult to reproduce in C57BL/6J mice: mice either show no lever-CS-directed behavior (Zweifel et al., 2009; Parker et al., 2011) or only demonstrate conditional locomotion in the vicinity of the lever-CS (Gore and Zweifel, 2013). Sign tracking in the form of full lever actuations, however, has not been reported previously.

Of interest was the considerable individual variation in whether a sign-tracking or goal-tracking conditional response emerged. Mice generally began with goal tracking (presumably because of previous magazine training), but 20–25% then developed overt sign-tracking conditional responses, some without any appreciable accompanying goal tracking (see summary bias score distribution, Figure 4), ultimately pressing the lever several hundred times per session; others continued goal tracking, and others performed both behaviors. Given that the mice studied here are, at least within a genotype group, isogenic, this variation in response type suggests considerable influence of (unmeasured) environmental or other nonheritable genetic factors, as has been observed in rats (Lomanowska et al., 2011).

While the sign-tracking conditional responses measured here appeared to be less common than in published data on rat behavior, and its onset is likely delayed relative to rats as well, video observation of testing chambers indicated that the sign-tracking phenotype is very much present in mice: those that engaged in this behavior did it consistently and vigorously, engaging in the same rapid biting, gnawing, and invigorated approach and contact with the lever-CS reported in rats (Zener, 1937; Jenkins and Moore, 1973; Boakes, 1977; Tomie, 1996; Flagel et al., 2010). Behavior was many times observed to be intensely focused toward the lever-CS, expressed as stereotypic sniffing and various other interactions, which did not necessarily result in a lever actuation; consequently, it is likely that mice sign track more often than we or others have reported. In addition, as has been repeatedly demonstrated in the rat (Robinson and Flagel, 2009; Flagel et al., 2011; Lomanowska et al., 2011), responding for conditioned reinforcement was higher in mice that sign tracked than in mice that goal tracked, indicating that similar phenotypic covariations exist across both species.

### Limitations

Although previous studies have, we did not demonstrate recombination of NR1 in dopamine neurons or measure phasic dopamine release here, and, consequently, some caution must be taken in the interpretation of the present findings (Engblom et al., 2008; Zweifel et al., 2008, 2009, 2011; Luo et al., 2010; Parker et al., 2010). Additionally, the finding of a DATcre × NR1 interaction for instrumental learning in the present study (i.e., both Cre recombinase and two floxed NR1 alleles were required to observe impairment) demonstrates that the model system functions as expected, at least in the context of instrumental reward learning.

Critically, as mentioned previously, the conditional inactivation of NR1 blocks NMDAR currents, which reduces phasic firing, but it also eliminates NMDAR-mediated synaptic plasticity (Engblom et al., 2008; Zweifel et al., 2008; Luo et al., 2010). This presents considerable difficulties with respect to interpreting our results strictly from the perspective of phasic dopamine release. Thus, although this study is not equipped to fully rule out a role for phasic dopamine release in conditional responses during pavlovian approach, we can conclude that they do not rely upon NMDAR-related plasticity in dopamine neurons or NMDAR-mediated components phasic activity, irrespective of whether it takes the form of goal tracking or sign tracking. More work is needed to fully ascribe the present results to differences in phasic dopaminergic neuron activity.

Because the transgenic mouse model used here is a constitutive knock-out, developmental alterations may have influenced the observed results. ROSA26-LacZ recombination is observable in the DATcre mouse used from E15 onward, and although no changes in DAT protein or D1 or D2 mRNA levels are observed (Bäckman et al., 2006), AMPA currents appear to be upregulated in a similar mouse line lacking NMDAR in dopamine neurons (Engblom et al., 2008; Zweifel et al., 2008). Little other work has been done regarding compensatory alterations in this mouse model, therefore, this remains an interpretational limitation.

Finally, our study lacked secondary confirmation of results to increase the confidence ascribed to the null results in the sign-tracking/goal-tracking experiment (e.g., a subthreshold dose of an NMDA antagonist to mimic the 30% loss of phasic release); future studies are needed to address this limitation.

### Conclusions

Here, we utilized a mouse model of compromised NMDA-dependent dopamine activity to characterize multiple components of reward-driven associative learning. Complementing the temporal precision of the optogenetics approaches, this approach allowed us to study the behavioral impact of putatively dampened endogenously generated phasic dopamine signals and loss of NMDAR-related synaptic plasticity. Our data revealed a clear role of NMDA activity in dopamine neurons in the acquisition of instrumental learning. We then tested causally, for the first time, predictions about the role of phasic dopamine in reward learning made by the incentive salience that contrast with those made by prediction error accounts. Though dopamine voltammetry data indicated a relationship between elevated CS-evoked dopamine activity and sign-tracking behavior (Flagel et al., 2011), our results, though not without notable interpretational limitations, lend no support to the conclusion of a causal relationship: the expression of conditional responses, regardless of whether they took the form of goal tracking or sign tracking, was unaffected in a model of eliminated NMDAR activity and putatively diminished NMDA-dependent phasic dopamine release. Thus, conditional responses associated with incentive salience attribution may not be under direct influence of the magnitude of NMDAR-regulated stimulus-evoked phasic release.

Therefore, our results are not fully consistent with the incentive salience perspective of phasic dopamine. They also are not necessarily uniformly consistent with a prediction error account of dopamine because putatively diminished phasic dopamine release did not affect pavlovian approach learning in any measured outcome. Thus, our results may be more congruent with a multifaceted conceptualization of dopaminergic transmission, wherein the behavioral significance of phasic dopamine could shift adaptively between prediction error, incentive salience attribution, and other forms of behavioral invigoration and flexibility, or combinations thereof, depending on the particular configuration of biological demands, internal goal states, and motivators present in the environment. This view is consistent with traditional views of dopamine as a neuromodulator, interacting with and adjusting ongoing circuit activity in a manner that can give rise to a multiplicity of context-dependent behavioral phenomena.

## Synthesis

The decision was a result of the Reviewing Editor Karen Szumlinski and the peer reviewers coming together and discussing their recommendations until a consensus was reached. A fact-based synthesis statement explaining their decision and outlining what is needed to prepare a revision is listed below. The following reviewers agreed to reveal their identity: Mary Torregrossa

### Synthesis of Reviews:

The report, entitled "Compromised NMDA/glutamate receptor expression in dopaminergic neurons impairs instrumental learning, but not Pavlovian goal-tracking or sign-tracking" was deemed unacceptable in its present state, following consultation with 2 reviewers with expertise in behavioral pharmacology and animal models of reinforcement. Both reviewers felt that the manuscript was well-prepared and well-written, but both cited the presentation of the data as box plots to be obscure and requested modifications to the data presentation to be more consistent with the extant literature. Both reviewers also had a number of interpretational issues that reduced enthusiasm for the report as currently written. Although the reviewers did find that the main conclusion that there is no straightforward relationship between dopamine release and error prediction or incentive salience consistent with the findings of the study, methodological weaknesses related to the use of Cre/loxP approaches to study the role of NR1 knock-down in DAT-expressing neurons were noted as not being fully integrated into the Discussion of the report that included: a lack of evidence for reduced NR1 expression (although it was noted that this was provided in the literature), a lack of evidence for changes in DA turn-over in cortico-limbic regions in mutant animals, a lack of consideration that NR1 knock down in dopamine neurons is likely to impair NMDA-mediated signaling/plasticity and not just phasic dopamine release to impact learning, lack of evidence that phasic dopamine release was normal in heterozygous controls (which could account for the low level of sign-tracking , and lack of genotypic differences, in sign-tracking reported), and interpretational confounds related to non-dopamine effects of Cre+ or floxed NR1. Both reviewers acknowledge that while a follow-up study of phasic dopamine release (or in the very least, an assay of dopamine turnover within ventral striatum or prefrontal cortex) would be greatly informative, at minimum, if the authors could revise their Discussion section to more fully integrate methodological limitations/interpretational confounds, then the report would be more improved. Having consulted with both reviewers on this report, I am of the opinion that this report either requires additional experimentation to either more tightly link phasic dopamine release to the learning phenomena under investigation or, in the very least, a major revision of the report, to include a down-play of the topic of phasic dopamine release and a greater focus on the role for NR1/NMDA on dopamine neurons behavior. Thus, we are all in agreement that major revisions are required on this report in order to render it more acceptable for publication in eNeuro.

### Reviewer 1:

In the manuscript titled, "Compromised NMDA/glutamate receptor expression in dopaminergic neurons impairs instrumental learning, but not Pavlovian goal-tracking or sign-tracking", the authors describe a series of studies evaluating the behavior of transgenic mice created to specifically lack the NMDA receptor obligatory subunit, NR1, in midbrain dopaminergic neurons. The authors found that these mice were largely normal in most assessments, including locomotor activity and appetitive Pavlovian learning including likelihood of developing goal-tracking vs. sign-tracking phenotypes. Mice lacking NR1 in DAT containing cells did have delayed acquisition of appetitive instrumental responding.

Overall, the experiments have the potential to be very informative about the role of DA neurons in appetitive learning paradigms, which could have a big impact on the field. The manuscript is generally well written, but I have several concerns that dampen my enthusiasm for the manuscript in its present form. My specific comments are listed below.

1. The authors make the claim that there is a "lack of phasic dopamine transmission" that mediates the observed acquisition defect and that phasic dopamine is not required in the other appetitive conditioning paradigms. However, the authors did not demonstrate that their mouse model 1) effectively decreased NR1 expression in DAT or TH positive cells (though this has been published), or 2) that phasic dopamine release was actually reduced in striatal regions in their mice. The dopamine utilization experiment in the ventral striatum is not informative, and suggests that the authors hypothesized that phasic dopamine in the nucleus accumbens is critical for appetitive conditioning; however, to my knowledge, reductions in phasic dopamine release in this mouse model have only been reported in the dorsal striatum. In addition, the degree to which phasic dopamine release is impaired depends on the stimulation intensity, with very little deficiency observed at stimulations of short duration with low to medium intensity. Therefore, the authors cannot conclude that a reduction in phasic dopamine has anything to do with the behavioral effects. If the author's show that the mice indeed lack NR1 subunits in dopaminergic neurons in the midbrain, then the authors could say that NR1 containing NMDA receptor transmission in midbrain DA neurons is not necessary for Pavlovian goal-tracking behavior, etc. The authors would need to conduct in vivo voltammetry experiments during the behavioral tasks to determine if any deficit in dopamine transmission in the nucleus accumbens really exists.

2. In addition, previous studies indicate that DAT is not expressed to a high degree in DA neurons projecting to the BLA or hippocampus. Normal BLA DA-ergic transmission could possibly explain the lack of effect on Pavlovian conditioning and responding for conditioned reinforcement. The authors should address this possibility.

3. In addition to the authors providing no evidence of DA transmission deficits in the homozygous mice, the authors did not demonstrate that there was a lack of a DA deficit in the heterozygous mice. In the experiments presented in figures 3-6, the authors' only control group is the heterozygous NR1 depletion mouse. While, it is unlikely that these mice have severe DA transmission deficits, it is possible that both the hets and homozygous have a loss of phasic DA, and without an appropriate control group either without Cre or without the floxed allele, it is impossible to determine if there is really a null effect, or a deficit in both experimental groups. The very low levels of observed sign-tracking in these experiments could conceivably be explained by DA signaling deficits in both groups.

4. In the results for the experiment presented in figure 2, the authors did not report a statistical difference between the DAT-NR1 knock outs and the Cre- floxed controls. Did this control group have normal NR1 expression in the brain? If so, then could the behavioral results simply be explained by the presence of 2 floxed NR1 alleles that somehow disrupt something else related to neuronal signaling. I don't know of specific examples where the presence of a floxed allele in the absence of Cre has an effect, but I do know of Cre+ mouse lines that have severe deficits in the expression of proteins in the cells in which they are expressed at baseline, without crossing to a floxed mouse. So, it's certainly possible that there are other things going on the neurons with Cre present or floxed NR1 that are not related to phasic dopamine output. The interpretation of this data should be done very carefully.

5. Despite all of the potential experimental confounds that should be addressed, the results presented are generally in line with reports from a previous study (Chen et al., 2008, Neuron 29: 288-297) which found that only cocaine, but not natural reward, induced plasticity in VTA synaptic function that was dependent on NR1 in dopamine neurons. The authors should discuss this previous paper in the context of their own work, and acknowledge that previous studies have found little differences in these mice when responding for food rewards.

6. The authors should clarify in the methods that the number of mice listed for each experiment includes all of the genotypes.

7. Knock out is spelled wrong in all of the figures.

8. The manuscript contains several typos throughout that should be corrected (e.g., line 91 quantiative should be quantitative).

9. Personally, I find presenting locomotor and acquisition data in box plot form more confusing than helpful. Perhaps line graphs with confidence intervals would make it easier for the reader to understand the data.

### Reviewer 2:

This ms reports the results of experiments that aimed to examine the involvement of NMDA-receptor dependent phasic dopamine release in instrumental learning and Pavlovian sign-tracking and goal-tracking behaviors, thereby testing leading hypotheses regarding the role of phasic dopamine in prediction error signaling and incentive salience. The manuscript has been carefully prepared. The design is clear and thorough, and the statistical approach and data presentation are proper (albeit somewhat obscure to most readers in the field). The main conclusion that there is not a straightforward relationship between phasic dopamine release and error prediction or incentive salience appears to be consistent with the findings.

Some methodological limitations weaken the authors' conclusions. First, NR1 were knocked down in DAT-expressing neurons using Cre/loxP technique. The resulting knock-out animals may have developmental adaptations. The authors investigated several possibilities in this respect, including behavioral abnormalities and possible changes in dopamine turnover in the striatum. Nevertheless, it would have been of great interest to determine possible alterations in dopamine utilization in relevant cortico-limbic brain regions. Second, as the authors noted, knock outs exhibited 30% of residual phasic dopamine release. In order to increase confidence in the null effects obtained in the study, it would have been important to supplement the current genetic approach (i.e., sub-threshold dose of NMDA antagonist, with the limitation that non-dopaminergic neurons are possibly affected). Third, and most importantly, NR1 knock down in dopamine neurons impairs a variety of NMDA-mediated plasticity events in addition to attenuating phasic dopamine release; therefore, it is not possible to ascribe the observed learning deficits to the latter. While each of these limitations are noted by the authors, their implications are not fully integrated adequately into the interpretation of the findings. Doing so will be important. And, while the resulting manuscript may lead to more questions than answers, it will remain an important addition to the literature.

March 25, 2015

Dear Dr. Szumlinski:

We appreciate the thorough and constructive reviews of our submitted manuscript (Compromised NMDA/glutamate receptor expression in dopaminergic neurons impairs instrumental learning, but not Pavlovian goal-tracking or sign-tracking eN-NWR-0040-14).

With regard to the concerns of the reviewers, we have made a series of modifications, all of which have been described in detail below and are highlighted in the text. We hope these modifications render our manuscript suitable for publication in the eNeuro.

### Reviewer #1:

1) However, the authors did not demonstrate that their mouse model 1) effectively decreased NR1 expression in DAT or TH positive cells (though this has been published), or 2) that phasic dopamine release was actually reduced in striatal regions in their mice.

This is a valid criticism. As the reviewer notes, we reasoned that the historical use of this (and related) mouse models had already demonstrated successful recombination of NR1 in dopamine cells (Engblom et al., 2008; Zweifel et al., 2008) and associated decrements in burst activity / phasic dopamine release (Zweifel et al., 2009; Parker et al., 2010; Zweifel et al., 2011) (Luo et al., 2011). In the same double transgenic line, PCR analysis indicated NR1 is deleted in the substantia nigra and ventral tegmental area (SN/VTA), and PCR-based detection of NR1 indicated deletion was specific to cells that were TH/DAT immunoreactive, but did not occur in non-immunoreactive cells gathered from the SN/VTA.

The following has been added to the limitations section:

Here, we did not demonstrate recombination of NR1 in dopamine neurons or measure phasic dopamine release, and consequently, some caution must be taken in the interpretation of the present findings, though previous studies have (Engblom et al., 2008; Zweifel et al., 2008; Zweifel et al., 2009; Parker et al., 2010; Luo et al., 2011; Zweifel et al., 2011), Additionally, the finding of a DATcre x NR1 interaction for instrumental learning in the present study (i.e., both Cre recombinase and two floxed NR1 alleles were required to observe impairment) demonstrates that the model system functions as expected, at least with regard to instrumental reward learning.

2) The dopamine utilization experiment in the ventral striatum is not informative, and suggests that the authors hypothesized that phasic dopamine in the nucleus accumbens is critical for appetitive conditioning; however, to my knowledge, reductions in phasic dopamine release in this mouse model have only been reported in the dorsal striatum.

We apologize for the unclear manner in which the dopamine utilization results were presented; these data were intended to demonstrate that the loss of NMDARs in dopamine neurons in the mouse model did not result in dramatic alterations to baseline function of the dopamine system; we do note that levels of DOPAC in the ventral striatum, a presumed measure of dopamine turnover, are lower in mutants than controls, generally supporting the idea that on-going DA transmission is modulated in some manner in the NR1 deficient animal. Moreover, recent data suggests that dopamine in the ventral striatum, rather than the dorsal striatum, is crucial for acquisition of Pavlovian approach responses (Darvas et al., 2014).

2) ...the authors cannot conclude that a reduction in phasic dopamine has anything to do with the behavioral effects. If the author's show that the mice indeed lack NR1 subunits in dopaminergic neurons in the midbrain, then the authors could say that NR1 containing NMDA receptor transmission in midbrain DA neurons is not necessary for Pavlovian goal-tracking behavior, etc.

We agree that there are multiple possible explanations for the behavioral effects observed and that not having measured NR1 or monitored dopamine limits our ability to claim one or the other. We certainly agree that a de-emphasis of explanations of the data with regard to dopamine activity is necessary, and we have correspondingly altered the discussion in multiple places to emphasize the NMDA receptor - relying on past literature regarding successful recombination (e.g., replaced "model system of attenuated phasic dopamine release" with "loss of NMDAR-mediated transmission in dopamine neurons" or "loss of NMDA-regulated dopamine activity"; added "putatively diminished" in the few places phasic dopamine is mentioned) - as well as added an additional line to the limitations section acknowledging that lower NR1 expression in dopamine neurons was not demonstrated within our study.

For example, in addition to the text added in 1), the following has been added to the discussion, incorporating other possible explanations for the data:

This result suggests a causal role for NMDA-mediated neurotransmission in dopamine neurons in instrumental learning. One possibility is that the loss of NMDA receptors disables one mechanism that contributes to phasic, stimulus-related dopamine neuron firing (Suaud-Chagny et al., 1992; Zweifel et al., 2009; Parker et al., 2010). Additionally, loss of the NMDAR eliminates NMDAR-mediated synaptic plasticity within dopamine neurons (Engblom et al., 2008; Zweifel et al., 2008; Luo et al., 2011). Thus, while the behavioral effect observed may well relate to altered phasic release, it is possible that other mechanisms are at play, including loss of synaptic plasticity between glutamatergic inputs and dopamine neurons or other downstream molecular changes. Because we did not measure NMDA expression or monitor dopamine activity in the context of behavior, it is difficult to disentangle these different interpretations, and further experiments are needed to parse these possibilities.

And the following to the discussion of the sign-tracking data:

Alternatively, it is possible that a loss of NMDAR-mediated synaptic plasticity or other NMDAR-dependent physiological mechanisms in mice lacking NMDAR in dopamine neurons obscured the observation of a behavioral difference in sign tracking.

The abstract has been reworded for a greater emphasis on NMDA activity:

These data support the view that reductions in NMDAR-signaling in dopamine neurons affect instrumental reward-related learning but do not lend support to hypotheses that suggest that the behavioral significance of this signaling includes incentive salience attribution.

2) In addition, previous studies indicate that DAT is not expressed to a high degree in DA neurons projecting to the BLA or hippocampus. Normal BLA DA-ergic transmission could possibly explain the lack of effect on Pavlovian conditioning and responding for conditioned reinforcement. The authors should address this possibility.

A study using the same double transgenic mouse model we employed investigated this question. The authors used PCR to detect recombination of NR1 in SN/VTA cells which were immunoreactive for TH but not DAT; successful recombination of NR1 was observed in 34 of 36 of such cells, indicating this mouse model likely does lack NR1 expression in TH+ cells even when they express relatively little DAT, likely including those that project to the BLA (Luo et al., 2011). This finding has been added to the discussion:

A final limitation is that these behaviors may be supported by dopaminergic projections to the basolateral amygdala or prefrontal cortex, as these cells express very little DAT (Lammel et al., 2008) and therefore NR1 recombination may have not fully occurred. However, a study using PCR to detect recombination of NR1 in the SN/VTA in the same mouse model found successful recombination of NR1 in 34 of 36 of such cells (Luo et al., 2011). Thus, Cre recombinase expression appears to be sufficient to drive excision of NR1 in the majority of dopaminergic neuronal populations, even those expressing very low levels of DAT.

3) ...while, it is unlikely that these mice have severe DA transmission deficits, it is possible that both the hets and homozygous have a loss of phasic DA, and without an appropriate control group either without Cre or without the floxed allele, it is impossible to determine if there is really a null effect, or a deficit in both experimental groups

We agree that the reviewer's criticism reflects a significant limitation in the degree to which the null effect can be interpreted. In the original manuscript, we did not show a third genotype group of mice that we tested (DATcre-;NR1flox/wt) because the box plots graphs were already difficult to read when they displayed the 2, key groups. We have now added statistics to the sign-tracking / goal-tracking results that support the conclusion that DATcre-;NR1flox/wt mice do not differ from DATcre+;NR1flox/wt mice, and therefore the latter group is suitable for primary comparisons to full knockouts.

4) In the results for the experiment presented in figure 2, the authors did not report a statistical difference between the DAT-NR1 knock outs and the Cre- floxed controls. Did this control group have normal NR1 expression in the brain? If so, then could the behavioral results simply be explained by the presence of 2 floxed NR1 alleles that somehow disrupt something else related to neuronal signaling. I don't know of specific examples where the presence of a floxed allele in the absence of Cre has an effect, but I do know of Cre+ mouse lines that have severe deficits in the expression of proteins in the cells in which they are expressed at baseline, without crossing to a floxed mouse. So, it's certainly possible that there are other things going on the neurons with Cre present or floxed NR1 that are not related to phasic dopamine output.

We apologize that this result was not made clearer in the figure caption. The difference between DAT-NR1 knock outs and Cre- floxed controls is indicated at the end of the caption as "# p < 0.05 vs. DATcre-;NR1flox/flox mice." Exploring the omnibus DATcre x NR1 x day interaction, within NR1flox/flox mice, we found a DATcre x day interaction; DATcre+;NR1flox/flox mice earned significantly less reinforcers than DATcre-;NR1flox/flox mice on day 5, and a similar trend was found on day 6 (see Results). With respect to interpretation of the data, we argue that the finding of a DATcre x NR1 interaction - which was the result of exclusively the knock outs performing worse than the other groups, indicating that both the Cre allele and both floxed alleles were required to produce the behavioral effect - makes the conclusion that the behavioral effect observed is the result genetic excision of NR1 from dopamine neurons most parsimonious. Note that statistical tests did not reveal any significant differences in instrumental performance amongst the three control groups; thus, in order to reduce the number of statistical comparisons made, we did not make comparisons between DATcre+;NR1flox/flox mice and DATcre-;NR1flox/wt mice. For clarity, we have we changed the caption to:

# p < 0.05 DATcre+;NR1flox/flox vs. DATcre-;NR1flox/flox mice.

With respect to the effect of the presence of the cre gene having an effect on DAT protein expression, initial characterization of this mouse indicated that there was on effect of carrying a single copy of the DATcre+ gene on DAT, and no effect on levels of D1 and D2 mRNA (Backman et al., 2006); all DATcre+ animals in the present study also carried only one copy. The similar levels of DAT expression to controls is likely owed to the internal ribosomal entry site facilitating bicistronic expression of DAT and cre. This has been noted in the limitations section of the discussion:

Because the transgenic mouse model used here is a constitutive knockout, developmental alterations may have influenced the observed results. LacZ recombination is observable in the DATcre mouse used from E15 onwards, and although no changes in DAT protein or D1 or D2 mRNA levels are observed (Backman et al., 2006)...

5) Despite all of the potential experimental confounds that should be addressed, the results presented are generally in line with reports from a previous study (Chen et al., 2008, Neuron 29: 288-297) which found that only cocaine, but not natural reward, induced plasticity in VTA synaptic function that was dependent on NR1 in dopamine neurons. The authors should discuss this previous paper in the context of their own work, and acknowledge that previous studies have found little differences in these mice when responding for food rewards.

Thank you for bringing this oversight to our attention. We agree that there is a wealth of data indicating drugs of abuse induce NMDA-related plasticity in the VTA; we do note that there are examples of such plasticity mechanisms occurring during appetitive behavior in putative midbrain dopamine neurons (Stuber et al., 2008). We have integrated a discussion of this finding within the context of our results in the discussion section:

Previous work has demonstrated persistent elevations in synaptic AMPA/NMDA ratios in dopamine neurons of animals self-administering cocaine, while AMPA/NMDA ratios were only transiently elevated in animals responding for food (Chen et al., 2008). NMDAR dynamics are therefore susceptible to modulation by rewarding experiences and reward modality. Thus, our observation that the acquisition and performance of Pavlovian conditional responses were not different in mice lacking NMDAR in dopamine neurons may depend upon the specific experimental conditions used here. We note, however, that enhanced AMPA/NMDA ratios have been observed during Pavlovian conditioning for food (Stuber et al., 2008), and in a similar mouse model of loss of NMDARs in dopamine neurons, cue-based learning was impaired (Zweifel et al., 2009). Conversely, the acquisition of a Pavlovian conditioned place preference for cocaine is unaffected in knockout mice (Engblom et al., 2008; Luo et al., 2011 but see Zweifel et al., 2008). Together, these data do not support the simple idea that NMDAR are involved in Pavlovian responses to drugs but not food.

6) The authors should clarify in the methods that the number of mice listed for each experiment includes all of the genotypes.

This has been corrected, with the numbers of mice in each genotype group, for each experiment, added to the methods.

7) Knock out is spelled wrong in all of the figures.

Thank you; this has been corrected.

8) The manuscript contains several typos throughout that should be corrected (e.g., line 91 quantiative should be quantitative).

Thank you again. We have reviewed the manuscript for typos.

9) Personally, I find presenting locomotor and acquisition data in box plot form more confusing than helpful. Perhaps line graphs with confidence intervals would make it easier for the reader to understand the data.

We agree, though we carefully followed journal instructions when preparing the original figures. For clarity, we have changed figures that depict acquisition of instrumental and Pavlovian behaviors, along with the locomotor data, to line graphs with standard error bars.

### Reviewer #2

1) ...NR1 were knocked down in DAT-expressing neurons using Cre/loxP technique. The resulting knock-out animals may have developmental adaptations. The authors investigated several possibilities in this respect, including behavioral abnormalities and possible changes in dopamine turnover in the striatum. Nevertheless, it would have been of great interest to determine possible alterations in dopamine utilization in relevant cortico-limbic brain regions.

The reviewer is correct in stating that developmental adaptations may have occurred in the mouse model employed; lacZ staining can be observed from E15 onwards (Backman et al., 2006). Indeed, increased AMPA currents have been observed in animals lacking NR1 in dopamine neurons, whether using a constitutive knockout or induced by viral infusion (Engblom et al., 2008; Zweifel et al., 2008); nevertheless, phasic release / burst firing activity is attenuated.

Though not shown in the manuscript, we also quantified monoamine levels in the medial prefrontal cortex; here, no changes DOPAC or DOPAC/DA levels were found. Unfortunately, we have no further data to add regarding developmental abnormalities.

As noted above, the following has been added to the limitations section:

Because the transgenic mouse model used here is a constitutive knockout, developmental alterations may have influenced the observed results. LacZ recombination is observable in the DATcre mouse used from E15 onwards, and although no changes in DAT protein or D1 or D2 mRNA levels are observed (Backman et al., 2006), AMPA currents appear to be upregulated in a similar mouse line lacking NMDAR in dopamine neurons (Engblom et al., 2008; Zweifel et al., 2008). Little other work has been done regarding compensatory alterations in this mouse model and therefore this remains an interpretational limitation.

2) ...as the authors noted, knock outs exhibited 30% of residual phasic dopamine release. In order to increase confidence in the null effects obtained in the study, it would have been important to supplement the current genetic approach (i.e., sub-threshold dose of NMDA antagonist, with the limitation that non-dopaminergic neurons are possibly affected).

We agree that supplementing our study with a secondary confirmatory approach would have made for a stronger dataset. We treated the 30% reduction as a strength of this mouse model, given the quantitative increase in CS-evoked dopamine in sign-trackers (Flagel et al., 2011); a sub-threshold pharmacological manipulation as outlined by the reviewer would, however, have been informative. We do note that one study using the same mouse model found no changes in sensitization to cocaine, intra-VTA infusion of an NMDA antagonist still blocked sensitization in knockout mice (Luo et al., 2011). While there was no attempt to use a sub-threshold dose, this result illustrates the degree to which non-dopaminergic cells within the VTA regulate behavior (which is informative in its own right). The absence of a supplemental approach to increase confidence in the negative results presented in the study is an important limitation, and is now noted as such in the limitations section of the manuscript:

Finally, our study lacked secondary confirmation of results in order increase the confidence ascribed to the null results in the sign-tracking / goal-tracking experiment (e.g., a sub-threshold dose of an NMDA antagonist to mimic the 30% loss of phasic release); future studies are needed to address this limitation

3) ...most importantly, NR1 knock down in dopamine neurons impairs a variety of NMDA-mediated plasticity events in addition to attenuating phasic dopamine release; therefore, it is not possible to ascribe the observed learning deficits to the latter. While each of these limitations are noted by the authors, their implications are not fully integrated adequately into the interpretation of the findings.

Thank you. We agree that we cannot ascribe our results fully to attenuated phasic dopamine release when plasticity mechanisms have also been disrupted. We have altered the discussion to more fully incorporate this interpretational limitation and have backed off the degree to which we attribute results to phasic release mechanisms in an attempt to yield a more balanced and cautious discussion of the data.

All of these suggestions have led to targeted modifications that we feel strengthen our manuscript. We hope that you judge our changes to be responsive and that the manuscript is now acceptable for publication in eNeuro.

Backman CM, Malik N, Zhang Y, Shan L, Grinberg A, Hoffer BJ, Westphal H, Tomac AC (2006) Characterization of a mouse strain expressing Cre recombinase from the 3' untranslated region of the dopamine transporter locus. Genesis 44:383-390.

Chen BT, Bowers MS, Martin M, Hopf FW, Gilroy AM, Carelli RM, Chou JK, Bonci A (2008) Cocaine but not natural reward self-administration nor passive cocaine infusion produces persistent LTP in the VTA. Neuron 59:288-297.

Darvas M, Wunsch AM, Gibbs JT, Palmiter RD (2014) Dopamine dependency for acquisition and performance of Pavlovian conditioned response. Proc Natl Acad Sci U S A 111:2764-2769.

Engblom D, Bilbao A, Sanchis-Segura C, Dahan L, Perreau-Lenz S, Balland B, Parkitna JR, Lujan R, Halbout B, Mameli M, Parlato R, Sprengel R, Luscher C, Schutz G, Spanagel R (2008) Glutamate receptors on dopamine neurons control the persistence of cocaine seeking. Neuron 59:497-508.

Flagel SB, Clark JJ, Robinson TE, Mayo L, Czuj A, Willuhn I, Akers CA, Clinton SM, Phillips PE, Akil H (2011) A selective role for dopamine in stimulus-reward learning. Nature 469:53-57.

Lammel S, Hetzel A, Häckel O, Jones I, Liss B, Roeper J (2008) Unique Properties of Mesoprefrontal Neurons within a Dual Mesocorticolimbic Dopamine System. Neuron 57:760-773.

Luo Y, Good CH, Diaz-Ruiz O, Zhang Y, Hoffman AF, Shan L, Kuang SY, Malik N, Chefer VI, Tomac AC, Lupica CR, Backman CM (2011) NMDA receptors on non-dopaminergic neurons in the VTA support cocaine sensitization. PloS one 5:e12141.

Parker JG, Zweifel LS, Clark JJ, Evans SB, Phillips PE, Palmiter RD (2010) Absence of NMDA receptors in dopamine neurons attenuates dopamine release but not conditioned approach during Pavlovian conditioning. Proc Natl Acad Sci U S A 107:13491-13496.

Stuber GD, Klanker M, de Ridder B, Bowers MS, Joosten RN, Feenstra MG, Bonci A (2008) Reward-predictive cues enhance excitatory synaptic strength onto midbrain dopamine neurons. Science 321:1690-1692.

Suaud-Chagny MF, Chergui K, Chouvet G, Gonon F (1992) Relationship between dopamine release in the rat nucleus accumbens and the discharge activity of dopaminergic neurons during local in vivo application of amino acids in the ventral tegmental area. Neuroscience 49:63-72.

Zweifel LS, Argilli E, Bonci A, Palmiter RD (2008) Role of NMDA receptors in dopamine neurons for plasticity and addictive behaviors. Neuron 59:486-496.

Zweifel LS, Fadok JP, Argilli E, Garelick MG, Jones GL, Dickerson TMK, Allen JM, Mizumori SJY, Bonci A, Palmiter RD (2011) Activation of dopamine neurons is critical for aversive conditioning and prevention of generalized anxiety. Nat Neurosci 14:620-626.

Zweifel LS, Parker JG, Lobb CJ, Rainwater A, Wall VZ, Fadok JP, Darvas M, Kim MJ, Mizumori SJ, Paladini CA, Phillips PE, Palmiter RD (2009) Disruption of NMDAR-dependent burst firing by dopamine neurons provides selective assessment of phasic dopamine-dependent behavior. Proc Natl Acad Sci U S A 106:7281-7288.

## References

[B1] Adamantidis AR, Tsai HC, Boutrel B, Zhang F, Stuber GD, Budygin EA, Touriño C, Bonci A, Deisseroth K, de Lecea L (2011) Optogenetic interrogation of dopaminergic modulation of the multiple phases of reward-seeking behavior. J Neurosci 31:10829-10835. 10.1523/JNEUROSCI.2246-11.2011 21795535 PMC3171183

[B2] Bäckman CM, Malik N, Zhang Y, Shan L, Grinberg A, Hoffer BJ, Westphal H, Tomac AC (2006) Characterization of a mouse strain expressing Cre recombinase from the 3' untranslated region of the dopamine transporter locus. Genesis 44:383-390. 10.1002/dvg.20228 16865686

[B3] Bean AJ, Roth RH (1991) Extracellular dopamine and neurotensin in rat prefrontal cortex *in vivo*: effects of median forebrain bundle stimulation frequency, stimulation pattern, and dopamine autoreceptors. J Neurosci 11:2694-2702. 1880545 10.1523/JNEUROSCI.11-09-02694.1991 PMC6575256

[B4] Berridge KC (2000) Reward learning: reinforcement, incentives, and expectations. In: The psychology of learning and motivation (Medin DL, ed) pp 223-278. New York: Academic.

[B5] Berridge KC (2007) The debate over dopamine's role in reward: the case for incentive salience. Psychopharmacology 191:391-431. 10.1007/s00213-006-0578-x 17072591

[B6] Berridge KC, Robinson TE (1998) What is the role of dopamine in reward: hedonic impact, reward learning, or incentive salience? Brain Re Brain Res Rev 28:309-369. 9858756 10.1016/s0165-0173(98)00019-8

[B7] Beutler LR, Wanat MJ, Quintana A, Sanz E, Bamford NS, Zweifel LS, Palmiter RD (2011) Balanced NMDA receptor activity in dopamine D1 receptor (D1R)- and D2R-expressing medium spiny neurons is required for amphetamine sensitization. Proc Natl Acad Sci U S A 108:4206-4211. 10.1073/pnas.1101424108 21368124 PMC3054029

[B8] Billstedt E (2000) Autism and Asperger syndrome: coexistence with other clinical disorders. Acta Psychiatr Scand 102:321-330. 11098802 10.1034/j.1600-0447.2000.102005321.x

[B9] Boakes R (1977) Performance on learning to associate a stimulus with positive reinforcement In: Operant-pavlovian interactions (Davis H, Hurwitz H, eds), pp 67-97. Hillsdale: Lawrence Erlbaum Associates.

[B10] Borgland SL, Malenka RC, Bonci A (2004) Acute and chronic cocaine-induced potentiation of synaptic strength in the ventral tegmental area: electrophysiological and behavioral correlates in individual rats. J Neurosci 24:7482-7490. 10.1523/JNEUROSCI.1312-04.2004 15329395 PMC6729639

[B11] Cagniard B, Beeler JA, Britt JP, McGehee DS, Marinelli M, Zhuang X (2006) Dopamine scales performance in the absence of new learning. Neuron 51:541-547. 10.1016/j.neuron.2006.07.026 16950153

[B12] Cannon CM, Palmiter RD (2003) Reward without dopamine. J Neurosci 23:10827-10831. 14645475 10.1523/JNEUROSCI.23-34-10827.2003 PMC6740991

[B13] Chen BT, Bowers MS, Martin M, Hopf FW, Guillory AM, Carelli RM, Chou JK, Bonci A (2008) Cocaine but not natural reward self-administration nor passive cocaine infusion produces persistent LTP in the VTA. Neuron 59:288-297. 10.1016/j.neuron.2008.05.024 18667156 PMC2593405

[B14] Crow TJ (1976) Specific monoamine systems as reward pathways In: Brain-stimulation reward (Wauquier A, Rolls ET, eds), pp 211-238. Amsterdam: North-Holland.

[B15] Day JJ, Roitman MF, Wightman RM, Carelli RM (2007) Associative learning mediates dynamic shifts in dopamine signaling in the nucleus accumbens. Nat Neurosci 10:1020-1028. 10.1038/nn1923 17603481

[B16] Doremus-Fitzwater TL, Spear LP (2011) Amphetamine-induced incentive sensitization of sign-tracking behavior in adolescent and adult female rats. Behav Neurosci 125:661-667. 21534648 10.1037/a0023763 PMC3144296

[B17] Engblom D, Bilbao A, Sanchis-Segura C, Dahan L, Perreau-Lenz S, Balland B, Parkitna JR, Luján R, Halbout B, Mameli M, Parlato R, Sprengel R, Lüscher C, Schütz G, Spanagel R (2008) Glutamate receptors on dopamine neurons control the persistence of cocaine seeking. Neuron 59:497-508. 10.1016/j.neuron.2008.07.010 18701074

[B18] Estes WK (1956) The problem of inference from curves based on group data. Psychol Bull 53:134-140. 13297917 10.1037/h0045156

[B19] Everitt BJ, Robbins TW (2005) Neural systems of reinforcement for drug addiction: from actions to habits to compulsion. Nat Neurosci 8:1481-1489. 10.1038/nn1579 16251991

[B20] Fiorillo CD, Tobler PN, Schultz W (2003) Discrete coding of reward probability and uncertainty by dopamine neurons. Science 299:1898-1902. 10.1126/science.1077349 12649484

[B21] Fitzpatrick CJ, Gopalakrishnan S, Cogan ES, Yager LM, Meyer PJ, Lovic V, Saunders BT, Parker CC, Gonzales NM, Aryee E, Flagel SB, Palmer AA, Robinson TE, Morrow JD (2013) Variation in the form of Pavlovian conditioned approach behavior among outbred male Sprague-Dawley rats from different vendors and colonies: sign-tracking vs. goal-tracking. PLoS One 8:e75042. 10.1371/journal.pone.0075042 24098363 PMC3787975

[B22] Flagel SB, Watson SJ, Robinson TE, Akil H (2007) Individual differences in the propensity to approach signals vs goals promote different adaptations in the dopamine system of rats. Psychopharmacology 191:599-607. 10.1007/s00213-006-0535-8 16972103

[B23] Flagel SB, Akil H, Robinson TE (2009) Individual differences in the attribution of incentive salience to reward-related cues: implications for addiction. Neuropharmacology 56 [Suppl 1 ]:139-148. 10.1016/j.neuropharm.2008.06.027 18619474 PMC2635343

[B24] Flagel SB, Robinson TE, Clark JJ, Clinton SM, Watson SJ, Seeman P, Phillips PE, Akil H (2010) An animal model of genetic vulnerability to behavioral disinhibition and responsiveness to reward-related cues: implications for addiction. Neuropsychopharmacology 35:388-400. 10.1038/npp.2009.142 19794408 PMC2794950

[B25] Flagel SB, Clark JJ, Robinson TE, Mayo L, Czuj A, Willuhn I, Akers CA, Clinton SM, Phillips PE, Akil H (2011) A selective role for dopamine in stimulus-reward learning. Nature 469:53-57. 10.1038/nature09588 21150898 PMC3058375

[B26] Forrest D, Yuzaki M, Soares HD, Ng L, Luk DC, Sheng M, Stewart CL, Morgan JI, Connor JA, Curran T (1994) Targeted disruption of NMDA receptor 1 gene abolishes NMDA response and results in neonatal death. Neuron 13:325-338. 8060614 10.1016/0896-6273(94)90350-6

[B27] Gallistel CR, Fairhurst S, Balsam P (2004) The learning curve: implications of a quantitative analysis. Proc Natl Acad Sci U S A 101:13124-13131. 10.1073/pnas.0404965101 15331782 PMC516535

[B28] Gonon FG (1988) Nonlinear relationship between impulse flow and dopamine released by rat midbrain dopaminergic neurons as studied by in vivo electrochemistry. Neuroscience 24:19-28. 10.1016/0306-4522(88)90307-7 3368048

[B29] Gore BB, Zweifel LS (2013) Genetic reconstruction of dopamine D1 receptor signaling in the nucleus accumbens facilitates natural and drug reward responses. J Neurosci 33:8640-8649. 10.1523/JNEUROSCI.5532-12.2013 23678109 PMC3684445

[B30] Grace AA, Bunney BS (1984) The control of firing pattern in nigral dopamine neurons: burst firing. J Neurosci 4:2877-2890. 6150071 10.1523/JNEUROSCI.04-11-02877.1984 PMC6564720

[B31] Groman SM, James AS, Jentsch JD (2009) Poor response inhibition: at the nexus between substance abuse and attention deficit/hyperactivity disorder. Neurosci Biobehav Rev 33:690-698. 10.1016/j.neubiorev.2008.08.008 18789354 PMC2728075

[B32] Haight JL, Flagel SB (2014) A potential role for the paraventricular nucleus of the thalamus in mediating individual variation in Pavlovian conditioned responses. Front Behav Neurosci 8:79. 10.3389/fnbeh.2014.00079 24672443 PMC3953953

[B33] Hnasko TS, Sotak BN, Palmiter RD (2005) Morphine reward in dopamine-deficient mice. Nature 438:854-857. 10.1038/nature04172 16341013

[B34] Iversen L, Iversen S, Bloom FE, Roth RH (2008) Introduction to neuropsychopharmacology. Oxford, UK: Oxford UP.

[B35] Jenkins HM, Moore BR (1973) The form of the auto-shaped response with food or water reinforcers. J Exp Anal Behav 20:163-181. 10.1901/jeab.1973.20-163 4752087 PMC1334117

[B36] Kalivas PW, Alesdatter JE (1993) Involvement of *N*-methyl-d-aspartate receptor stimulation in the ventral tegmental area and amygdala in behavioral sensitization to cocaine. J Pharmacol Exp Ther 267:486-495. 8229779

[B37] Kelley AE (2004) Ventral striatal control of appetitive motivation: role in ingestive behavior and reward-related learning. Neurosci Biobehav Rev 27:765-776. 10.1016/j.neubiorev.2003.11.015 15019426

[B38] Kim KM, Baratta MV, Yang A, Lee D, Boyden ES, Fiorillo CD (2012) Optogenetic mimicry of the transient activation of dopamine neurons by natural reward is sufficient for operant reinforcement. PLoS One 7:e33612. 10.1371/journal.pone.0033612 22506004 PMC3323614

[B39] Lammel S, Hetzel A, Häckel O, Jones I, Liss B, Roeper J (2008) Unique properties of mesoprefrontal neurons within a dual mesocorticolimbic dopamine system. Neuron 57:760-773. 10.1016/j.neuron.2008.01.022 18341995

[B40] Lashley KS (1942) An examination of the “continuity theory” as applied to discriminative learning. J Gen Psychol 26:241-265. 10.1080/00221309.1942.10545168

[B41] Ljungberg T, Apicella P, Schultz W (1992) Responses of monkey dopamine neurons during learning of behavioral reactions. J Neurophysiol 67:145-163. 1552316 10.1152/jn.1992.67.1.145

[B42] Lomanowska AM, Lovic V, Rankine MJ, Mooney SJ, Robinson TE, Kraemer GW (2011) Inadequate early social experience increases the incentive salience of reward-related cues in adulthood. Behav Brain Res 220:91-99. 10.1016/j.bbr.2011.01.033 21277909

[B43] Lowry OH, Rosebrough NJ, Farr AL, Randall RJ (1951) Protein measurement with the Folin phenol reagent. J Biol Chem 193:265-275. 14907713

[B44] Luo Y, Good CH, Diaz-Ruiz O, Zhang Y, Hoffman AF, Shan L, Kuang SY, Malik N, Chefer VI, Tomac AC, Lupica CR, Bäckman CM (2010) NMDA receptors on non-dopaminergic neurons in the VTA support cocaine sensitization. PLoS One 5:e12141. 10.1371/journal.pone.0012141 20808436 PMC2922329

[B45] Martin-Soelch C, Linthicum J, Ernst M (2007) Appetitive conditioning: neural bases and implications for psychopathology. Neurosci Biobehav Rev 31:426-440. 10.1016/j.neubiorev.2006.11.002 17210179 PMC2693132

[B46] Meyer PJ, Lovic V, Saunders BT, Yager LM, Flagel SB, Morrow JD, Robinson TE (2012) Quantifying individual variation in the propensity to attribute incentive salience to reward cues. PLoS One 7:e38987. 10.1371/journal.pone.0038987 22761718 PMC3382216

[B47] Nestler EJ, Carlezon WA Jr (2006) The mesolimbic dopamine reward circuit in depression. Biol Psychiatry 59:1151-1159. 10.1016/j.biopsych.2005.09.018 16566899

[B48] Neuringer A (2002) Operant variability: evidence, functions, and theory. Psychon Bull Rev 9:672-705. 12613672 10.3758/bf03196324

[B49] Olausson P, Jentsch JD, Tronson N, Neve RL, Nestler EJ, Taylor JR (2006) DeltaFosB in the nucleus accumbens regulates food-reinforced instrumental behavior and motivation. J Neurosci 26:9196-9204. 10.1523/JNEUROSCI.1124-06.2006 16957076 PMC6674495

[B50] Ostlund SB, LeBlanc KH, Kosheleff AR, Wassum KM, Maidment NT (2014) Phasic mesolimbic dopamine signaling encodes the facilitation of incentive motivation produced by repeated cocaine exposure. Neuropsychopharmacology 39:2441-249. 24804846 10.1038/npp.2014.96 PMC4138756

[B51] Overton PG, Clark D (1997) Burst firing in midbrain dopaminergic neurons. Brain Res Brain Res Rev 25:312-334. 9495561 10.1016/s0165-0173(97)00039-8

[B52] Parker JG, Zweifel LS, Clark JJ, Evans SB, Phillips PE, Palmiter RD (2010) Absence of NMDA receptors in dopamine neurons attenuates dopamine release but not conditioned approach during Pavlovian conditioning. Proc Natl Acad Sci USA 107:13491-13496. 10.1073/pnas.1007827107 20616081 PMC2922155

[B53] Parker JG, Beutler LR, Palmiter RD (2011) The contribution of NMDA receptor signaling in the corticobasal ganglia reward network to appetitive pavlovian learning. J Neurosci 31:11362-11369. 10.1523/JNEUROSCI.2411-11.2011 21813695 PMC3156031

[B54] Peciña S, Cagniard B, Berridge KC, Aldridge JW, Zhuang X (2003) Hyperdopaminergic mutant mice have higher "wanting" but not "liking" for sweet rewards. J Neurosci 23:9395-9402. 14561867 10.1523/JNEUROSCI.23-28-09395.2003 PMC6740586

[B55] Perez-Sepulveda JA, Flagel SB, Garcia-Fuster MJ, Slusky RJ, Aldridge JW, Watson S, Akil H (2013) Differential impact of a complex environment on positive affect in an animal model of individual differences in emotionality. Neuroscience 248:436-447. 10.1016/j.neuroscience.2013.06.015 23806722 PMC3841231

[B56] Ranaldi R, Kest K, Zellner MR, Lubelski D, Muller J, Cruz Y, Saliba M (2011) The effects of VTA NMDA receptor antagonism on reward-related learning and associated c-fos expression in forebrain. Behav Brain Res 216:424-432. 10.1016/j.bbr.2010.08.026 20801158

[B57] Redgrave P, Prescott TJ, Gurney K (1999) Is the short-latency dopamine response too short to signal reward error? Trends Neurosci 22:146-151. 10203849 10.1016/s0166-2236(98)01373-3

[B58] Rescorla RA, Wagner AR (1972) A theory of Pavlovian conditioning: variations in the effectiveness of reinforcement and nonreinforcement In: Classical conditioning II: current research and theory (Black AH, Prokasy WF, eds), pp 64-99. New York: Appleton-Century-Crofts.

[B59] Robbins TW (1978) The acquisition of responding with conditioned reinforcement: effects of pipradrol, methylphenidate, d-amphetamine, and nomifensine. Psychopharmacology 58:79-87. 27837 10.1007/BF00426794

[B60] Robbins TW, Everitt BJ (1992) Functions of dopamine in the dorsal and ventral striatum. Semin Neurosci 4:119-127. 10.1016/1044-5765(92)90010-Y

[B61] Robinson S, Sandstrom SM, Denenberg VH, Palmiter RD (2005) Distinguishing whether dopamine regulates liking, wanting, and/or learning about rewards. Behav Neurosci 119:5-15. 10.1037/0735-7044.119.1.5 15727507

[B62] Robinson TE, Berridge KC (1993) The neural basis of drug craving: an incentive-sensitization theory of addiction. Brain Res Brain Res Rev 18:247-291. 8401595 10.1016/0165-0173(93)90013-p

[B63] Robinson TE, Berridge KC (2000) The psychology and neurobiology of addiction: an incentive-sensitization view. Addiction 95 [Suppl 2 ]:S91-117. 11002906 10.1080/09652140050111681

[B64] Robinson TE, Flagel SB (2009) Dissociating the predictive and incentive motivational properties of reward-related cues through the study of individual differences. Biol Psychiatry 65:869-873. 10.1016/j.biopsych.2008.09.006 18930184 PMC2737368

[B65] Salamone JD (1994) The involvement of nucleus accumbens dopamine in appetitive and aversive motivation. Behav Brain Res 61:117-133. 8037860 10.1016/0166-4328(94)90153-8

[B66] Salamone JD, Wisniecki A, Carlson BB, Correa M (2001) Nucleus accumbens dopamine depletions make animals highly sensitive to high fixed ratio requirements but do not impair primary food reinforcement. Neuroscience 105:863-870. 11530224 10.1016/s0306-4522(01)00249-4

[B67] Salamone JD, Correa M, Mingote SM, Weber SM (2005) Beyond the reward hypothesis: alternative functions of nucleus accumbens dopamine. Curr Opin Pharmacol 5:34-41. 10.1016/j.coph.2004.09.004 15661623

[B68] Schultz W (2002) Getting formal with dopamine and reward. Neuron 36:241-263. 12383780 10.1016/s0896-6273(02)00967-4

[B69] Schultz W, Apicella P, Ljungberg T (1993) Responses of monkey dopamine neurons to reward and conditioned stimuli during successive steps of learning a delayed response task. J Neurosci 13:900-913. 8441015 10.1523/JNEUROSCI.13-03-00900.1993 PMC6576600

[B70] Schultz W, Dayan P, Montague PR (1997) A neural substrate of prediction and reward. Science 275:1593-1599. 9054347 10.1126/science.275.5306.1593

[B71] Shiflett MW, Balleine BW (2011) Molecular substrates of action control in cortico-striatal circuits. Prog Neurobiol 95:1-13. 10.1016/j.pneurobio.2011.05.007 21704115 PMC3175490

[B72] Soriano P (1999) Generalized lacZ expression with the ROSA26 Cre reporter strain. Nat Genet 21:70-71. 10.1038/5007 9916792

[B73] Steinberg EE, Keiflin R, Boivin JR, Witten IB, Deisseroth K, Janak PH (2013) A causal link between prediction errors, dopamine neurons and learning. Nat Neurosci 16:966-973. 10.1038/nn.3413 23708143 PMC3705924

[B74] Stuber GD, Klanker M, de Ridder B, Bowers MS, Joosten RN, Feenstra MG, Bonci A (2008) Reward-predictive cues enhance excitatory synaptic strength onto midbrain dopamine neurons. Science 321:1690-1692. 10.1126/science.1160873 18802002 PMC2613864

[B75] Suaud-Chagny MF, Chergui K, Chouvet G, Gonon F (1992) Relationship between dopamine release in the rat nucleus accumbens and the discharge activity of dopaminergic neurons during local in vivo application of amino acids in the ventral tegmental area. Neuroscience 49:63-72. 1357587 10.1016/0306-4522(92)90076-e

[B76] Sutton RS, Barto AG (1998) Reinforcement learning: an introduction. Cambridge, MA: MIT. 10.1109/TNN.1998.712192

[B77] Swerdlow NR, Koob GF (1987) Dopamine, schizophrenia, mania, and depression: toward a unified hypothesis of cortico-striatopallido-thalamic function. Behav Brain Sci 10:197-208. 10.1017/S0140525X00047488

[B78] Taylor JR, Jentsch JD (2001) Repeated intermittent administration of psychomotor stimulant drugs alters the acquisition of Pavlovian approach behavior in rats: differential effects of cocaine, d-amphetamine and 3,4- methylenedioxymethamphetamine (“Ecstasy”). Biol Psychiatry 50:137-143. 11526995 10.1016/s0006-3223(01)01106-4

[B79] Tindell AJ, Berridge KC, Zhang J, Peciña S, Aldridge JW (2005) Ventral pallidal neurons code incentive motivation: amplification by mesolimbic sensitization and amphetamine. Eur J Neurosci 22:2617-2634. 10.1111/j.1460-9568.2005.04411.x 16307604

[B80] Tomie A (1996) Locating reward cue at response manipulandum (CAM) induces symptoms of drug abuse. Neurosci Biobehav Rev 20:505-535. 8880737 10.1016/0149-7634(95)00023-2

[B81] Tonegawa S, Tsien JZ, McHugh TJ, Huerta P, Blum KI, Wilson MA (1996) Hippocampal CA1-region-restricted knockout of NMDAR1 gene disrupts synaptic plasticity, place fields, and spatial learning. Cold Spring Harb Symp Quant Biol 61:225-238. 9246451

[B82] Tsai HC, Zhang F, Adamantidis A, Stuber GD, Bonci A, de Lecea L, Deisseroth K (2009) Phasic firing in dopaminergic neurons is sufficient for behavioral conditioning. Science 324:1080-1084. 10.1126/science.1168878 19389999 PMC5262197

[B83] Tsien JZ, Huerta PT, Tonegawa S (1996) The essential role of hippocampal CA1 NMDA receptor-dependent synaptic plasticity in spatial memory. Cell 87:1327-1338. 8980238 10.1016/s0092-8674(00)81827-9

[B84] Ungless MA, Whistler JL, Malenka RC, Bonci A (2001) Single cocaine exposure in vivo induces long-term potentiation in dopamine neurons. Nature 411:583-587. 10.1038/35079077 11385572

[B85] Vanderschuren LJ, Kalivas PW (2000) Alterations in dopaminergic and glutamatergic transmission in the induction and expression of behavioral sensitization: a critical review of preclinical studies. Psychopharmacology 151:99-120. 10972458 10.1007/s002130000493

[B86] Verbeke G, Molenberghs G (2009) Linear mixed models for longitudinal data. New York: Springer.

[B87] Waelti P, Dickinson A, Schultz W (2001) Dopamine responses comply with basic assumptions of formal learning theory. Nature 412:43-48. 10.1038/35083500 11452299

[B88] Williams DR, Williams H (1969) Auto-maintenance in the pigeon: sustained pecking despite contingent non-reinforcement. J Exp Anal Behav 12:511-520. 16811370 10.1901/jeab.1969.12-511 PMC1338642

[B89] Wise RA, Rompre PP (1989) Brain dopamine and reward. Annu Rev Psychol 40:191-225. 10.1146/annurev.ps.40.020189.001203 2648975

[B90] Witten IB, Steinberg EE, Lee SY, Davidson TJ, Zalocusky KA, Brodsky M, Yizhar O, Cho SL, Gong S, Ramakrishnan C, Stuber GD, Tye KM, Janak PH, Deisseroth K (2011) Recombinase-driver rat lines: tools, techniques, and optogenetic application to dopamine-mediated reinforcement. Neuron 72:721-733. 10.1016/j.neuron.2011.10.028 22153370 PMC3282061

[B91] Wolf ME (1998) The role of excitatory amino acids in behavioral sensitization to psychomotor stimulants. Prog Neurobiol 54:679-720. 9560846 10.1016/s0301-0082(97)00090-7

[B92] Wolf ME, White FJ, Hu XT (1994) MK-801 prevents alterations in the mesoaccumbens dopamine system associated with behavioral sensitization to amphetamine. J Neurosci 14:1735-1745. 8126567 10.1523/JNEUROSCI.14-03-01735.1994 PMC6577589

[B93] Wyvell CL, Berridge KC (2000) Intra-accumbens amphetamine increases the conditioned incentive salience of sucrose reward: enhancement of reward "wanting" without enhanced “liking” or response reinforcement. J Neurosci 20:8122-8130. 11050134 10.1523/JNEUROSCI.20-21-08122.2000 PMC6772712

[B94] Wyvell CL, Berridge KC (2001) Incentive sensitization by previous amphetamine exposure: increased cue-triggered “wanting” for sucrose reward. J Neurosci 21:7831-7840. 11567074 10.1523/JNEUROSCI.21-19-07831.2001 PMC6762900

[B95] Yin HH, Zhuang X, Balleine BW (2006) Instrumental learning in hyperdopaminergic mice. Neurobiol Learn Mem 85:283-288. 10.1016/j.nlm.2005.12.001 16423542

[B96] Zener K (1937) The significance of behavior accompanying conditioned salivary secretion for theories of the conditioned response. Am J Psychol 50:384 10.2307/1416644

[B97] Zweifel LS, Argilli E, Bonci A, Palmiter RD (2008) Role of NMDA receptors in dopamine neurons for plasticity and addictive behaviors. Neuron 59:486-496. 10.1016/j.neuron.2008.05.028 18701073 PMC2556153

[B98] Zweifel LS, Parker JG, Lobb CJ, Rainwater A, Wall VZ, Fadok JP, Darvas M, Kim MJ, Mizumori SJ, Paladini CA, Phillips PE, Palmiter RD (2009) Disruption of NMDAR-dependent burst firing by dopamine neurons provides selective assessment of phasic dopamine-dependent behavior. Proc Natl Acad Sci USA 106:7281-7288. 10.1073/pnas.0813415106 19342487 PMC2678650

[B99] Zweifel LS, Fadok JP, Argilli E, Garelick MG, Jones GL, Dickerson TMK, Allen JM, Mizumori SJY, Bonci A, Palmiter RD (2011) Activation of dopamine neurons is critical for aversive conditioning and prevention of generalized anxiety. Nat Neurosci 14:620-626. 10.1038/nn.2808 21499253 PMC3083461

